# Impact of Tobacco Control Interventions on Smoking Initiation, Cessation, and Prevalence: A Systematic Review

**DOI:** 10.1155/2012/961724

**Published:** 2012-06-07

**Authors:** Lisa M. Wilson, Erika Avila Tang, Geetanjali Chander, Heidi E. Hutton, Olaide A. Odelola, Jessica L. Elf, Brandy M. Heckman-Stoddard, Eric B. Bass, Emily A. Little, Elisabeth B. Haberl, Benjamin J. Apelberg

**Affiliations:** ^1^Division of General Internal Medicine, Johns Hopkins University School of Medicine, Baltimore, MD 21205, USA; ^2^Department of Epidemiology, Institute for Global Tobacco Control, Johns Hopkins University Bloomberg School of Public Health, Baltimore, MD 21205, USA; ^3^Department of Health, Behavior and Society, Institute for Global Tobacco Control, Johns Hopkins University Bloomberg School of Public Health, Baltimore, MD 21205, USA; ^4^Department of Psychiatry and Behavioral Sciences, Johns Hopkins University School of Medicine, Baltimore, MD 21287, USA; ^5^Department of Internal Medicine, Albert Einstein Medical Center, Philadelphia, PA 19141, USA; ^6^Cancer Prevention Fellowship Program, Division of Cancer Prevention, National Cancer Institute, Bethesda, MD 20892, USA; ^7^Department of Health Policy and Management, Johns Hopkins University Bloomberg School of Public Health, Baltimore, MD, USA

## Abstract

*Background*. Policymakers need estimates of the impact of tobacco control (TC) policies to set priorities and targets for reducing tobacco use. We systematically reviewed the independent effects of TC policies on smoking behavior. *Methods*. We searched MEDLINE (through January 2012) and EMBASE and other databases through February 2009, looking for studies published after 1989 in any language that assessed the effects of each TC intervention on smoking prevalence, initiation, cessation, or price participation elasticity. Paired reviewers extracted data from studies that isolated the impact of a single TC intervention. *Findings*. We included 84 studies. The strength of evidence quantifying the independent effect on smoking prevalence was high for increasing tobacco prices and moderate for smoking bans in public places and antitobacco mass media campaigns. Limited direct evidence was available to quantify the effects of health warning labels and bans on advertising and sponsorship. Studies were too heterogeneous to pool effect estimates. *Interpretations*. We found evidence of an independent effect for several TC policies on smoking prevalence. However, we could not derive precise estimates of the effects across different settings because of variability in the characteristics of the intervention, level of policy enforcement, and underlying tobacco control environment.

## 1. Introduction

Tobacco smoking is one of the leading causes of preventable death, responsible for over 5 million deaths annually [1]. Currently, more than 1 billion people smoke, with over 80% living in low- and middle-income countries [2]. However, countries are at different stages of the tobacco epidemic [3]. Many countries have achieved substantial declines in smoking and tobacco-related disease through the implementation of comprehensive tobacco control programs, while others are experiencing increases in smoking prevalence. Tobacco control efforts have evolved over time as evidence has grown to support the use of different approaches. The population-based approaches most commonly used have included increased taxes, public education through mass media campaigns and health warnings, tobacco marketing restrictions, and the introduction of smoke-free indoor environments.

With the introduction of the World Health Organization's (WHO) Framework Convention on Tobacco Control (FCTC) [4] and MPOWER (Monitor, Protect, Offer, Warn, Enforce, Raise) policy package [5], tobacco control policies are being implemented worldwide. To model the impacts of these policies and develop achievable targets for smoking prevalence, policy makers need estimates of the independent effects of interventions on smoking behavior. We performed a systematic review to evaluate the independent effect on smoking prevalence of four tobacco control policies outlined in the WHO MPOWER Package [5]: increasing taxes on tobacco products, banning smoking in public places, banning advertising and sponsorship of tobacco products, and educating people through health warning labels and antitobacco mass media campaigns (Table 1). We focused on the degree of certainty in the estimated impact and factors that may influence the impact.

## 2. Methods

### 2.1. Study Design and Scope

For our systematic review of published studies, smoking was defined as the use of cigarettes and/or other smoked products, such as cigars, cigarillos, bidis, hookahs, water pipes, and kreteks. We excluded smokeless tobacco products. Outcomes of interest were smoking prevalence, initiation or cessation rates, and price participation elasticity (PPE) (the relative percentage change in smoking prevalence for every 1% change in price). We excluded outcomes such as quit attempts or tobacco consumption because they did not directly address the impact of interventions on smoking prevalence.

### 2.2. Search Strategy

We searched five databases: MEDLINE (accessed via PubMed, January 1950 through January 2012), EMBASE (January 1974 through February 2009), The Cochrane Library (Issue 1, 2009), the Cumulative Index to Nursing and Allied Health Literature (CINAHL, January 1982 through February 2009), and PsycInfo (from inception through February 2009). Our electronic search strategy used medical subject headings and text words for smoking and the tobacco control interventions and was limited to human subjects (see the appendix for the MEDLINE search string). We reviewed recent issues of ten economics and public health journals, reference lists of included articles, relevant reviews, books, and reports.

### 2.3. Study Selection

Two reviewers independently assessed titles, abstracts, and articles for inclusion. We included peer-reviewed studies published in any language that: measured smoking prevalence, initiation, cessation, or PPE; assessed the independent effects of at least one of the tobacco control interventions; met our study design criteria (Table 1). Because modeling approaches typically require estimates of independent effects, we excluded studies evaluating multicomponent interventions. Studies published prior to 1990 were excluded because the smoking population may have changed over time. Conflicts on eligibility were resolved through consensus.

### 2.4. Data Extraction

Reviewers used a Web-based system to extract data from eligible studies on study design, interventions, and smoking prevalence. Extracted data were checked by a second reviewer. Study quality was assessed independently by two reviewers.

We were unable to conduct meta-analyses because of the heterogeneity of the studies. Instead, we prepared a qualitative summary of results by intervention type and highlighted key sources of heterogeneity.

### 2.5. Grading of Evidence

We graded the quantity, quality, and consistency of results based on the GRADE working group criteria [6]. “High” strength of evidence indicates high confidence that the evidence reflects the true effect, and further research is very unlikely to change the result. “Moderate” strength of evidence indicates moderate confidence that the evidence reflects the true effect, and further research may change the result. “Low” strength of evidence indicates low confidence that the evidence reflects the true effect, and further research is likely to change the result. An “insufficient” grade indicates that no evidence was available to quantify the independent effect.

### 2.6. Role of the Funding Source

The International Union Against Tuberculosis and Lung Disease suggested the topic, but was not involved in the collection, analysis, and interpretation of the data, or in the writing of the paper. The authors retained full control over the conduct and reporting of the paper.

## 3. Results

### 3.1. Search Results

**Figure 1:**
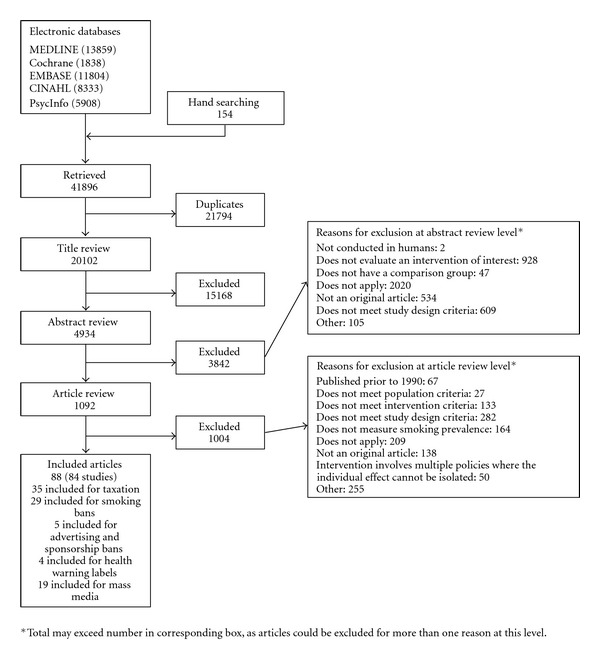
Summary of the literature search (number of articles).

From our search of 20,102 unique citations, we included 84 studies (88 publications) (Figure 1). Thirty-five evaluated taxation, 29 evaluated smoking bans, 5 evaluated advertising or sponsorship bans, 4 evaluated health warning labels, and 19 evaluated mass media campaigns. Twelve studies assessed smoking initiation (11 among youths), 25 assessed smoking cessation (4 among youths), and 52 (19 among youths) assessed smoking prevalence. Eight studies were conducted in low- and middle-income countries. The overall summary of the evidence for these interventions is presented in Table 2.

### 3.2. Increasing Taxes on Tobacco Products

We found high strength of evidence to quantify the impact of increases in tobacco pricing. The PPEs ranged from −1.41 to −0.10 (interpreted as a 1–14% decrease in smoking prevalence for every 10% increase in price) among youths and −0.45 to 0.10 among adults. The larger PPE for youths is consistent with prior evidence that young people are more price sensitive due to lower levels of disposable income.

#### 3.2.1. Youths

Five [7–11], one [12], and nine studies [13–21] evaluated the impact of increased taxes on smoking initiation, cessation, and prevalence among youths, respectively (Table 3). All but four [8, 15, 16, 19] were conducted in the US. One study was conducted among youths in 17 low- and middle-income countries [15]. Of the five studies examining smoking initiation, four found a statistically significant negative association with increasing taxes/prices (PPE for initiation ranged from −0.65 to −0.09) [7–10], while the other did not (PPE for initiation, −0.003) [11]. All nine studies evaluating youth smoking prevalence found a significant negative effect of taxes/prices, at least among a subset of their samples [13–21]. The study conducted among low- and middle-income countries reported a PPE for local brands of −0.74 and a PPE for foreign brands of −1.09 [15]. The study examining smoking cessation found a price elasticity of cessation of 1.15 among males and 1.17 among females [12].

#### 3.2.2. Adults

Six studies evaluated the impact of taxes/prices on smoking cessation among adults [12, 22–26]. Three found a statistically significant effect of taxes/price [12, 24, 25], while one found an impact only in the short term (4 months) [26]. One study found a significant association when evaluating prices, but not province-level taxes [22]. One study conducted in Mexico reported a 13% quit rate after a tax increase [23]. Twelve [25, 27–37] of 16 studies evaluating the effects of taxes/prices on adult smoking prevalence demonstrated a significant negative impact among at least a subset of their sample. Statistically significant effects of price/tax on smoking prevalence were consistently found in studies in high-income countries, such as the US [25, 31–33, 37], Australia [27, 30, 35], and Italy [34]. However, one study conducted in the European Union failed to find a correlation between cigarette affordability and smoking prevalence [38]. The results from low- and middle-income countries were more heterogeneous. Studies in South Africa and Russia found a significant decrease in smoking prevalence after a tax/price increase, with an estimated PPE of −0.30 and −0.10, respectively [29, 36]. A study in Mexico found a price elasticity of demand (i.e., the relative percentage change in demand for a 1% change in price) of −0.52, but the PPE was only −0.06 [39]. However, data on smoking participation was based on the purchasing patterns of all members of the household, meaning that an impact is only observed if all members of the household quit. A recent study in China [29] also found a relatively small PPE, which may be explained by the high level of affordability and the wide range of cigarette prices, which allows smokers to substitute a lower cost brand [40].

### 3.3. Banning Smoking in Public Places

We found moderate strength of evidence to quantify the impact of smoking bans. Twenty-nine studies measured the independent effect of smoking bans on initiation (2 studies), cessation (9 studies), and/or prevalence of smoking (20 studies). The strongest evidence was observed among studies of smoking prevalence, compared with studies assessing smoking initiation and cessation.

The studies that evaluated smoking initiation reported mixed results (Table 4) [41, 42].

Of the nine studies that evaluated smoking cessation, three had a concurrent comparison group [41, 43, 44]. Two studies found no significant association between the smoking ban and cessation rates (adjusted odds ratios ranging from 0.91 to 0.95) [43, 44], while the other found a significantly lower cessation rate (adjusted odds ratios ranging from 0.65 to 0.66) [41]. The other studies lacked a comparison group, making it difficult to draw conclusions. Four studies reported quit rates ranging from 5% to 15% [45–48], another reported a 5.1% increase in the quit rate in the 3-month period prior to the ban [49], and the other reported a 7.0% absolute difference in quit rates between those employed and those unemployed [50].

The effectiveness of a smoking ban likely depends on the comprehensiveness of legislation, level of enforcement, public support, and degree of prior legislation in place. Three studies evaluating a new, local, and comprehensive smoking ban reported the strongest effects on smoking prevalence [51–53]. In Saskatoon, Canada, smoking prevalence dropped from 24.1% to 18.2% one year after the ban [53]. In Lexington-Fayette County, Kentucky, smoking prevalence declined from 25.7% to 17.5% 20 months after the ban [52]. Another study conducted among college students in two different counties in Kentucky (Lexington-Fayette county and Louisville Metro) reported significant decreases in smoking prevalence 3.5 years (*P* = 0.005) and 8 months after their respective smoking bans [51]. However, a cohort study in Minnesota found no significant impact on smoking prevalence [54].

Studies conducted at the national level, where tobacco control activities have been ongoing tended to find less dramatic changes in smoking prevalence. For example, an Italian pre-/post- study without a comparison group found a significant decline in smoking prevalence among men (−8.5%, *P* < 0.05) and younger Italians (−7.4%, *P* < 0.05) following the introduction of a complete smoking ban [55]. In Spain, a study found a lower than expected smoking prevalence 1 year after the implementation of a partial smoking ban, but smoking prevalence returned to normal 3 years after the ban [56]. Similarly, a time series analysis in Scotland found a significant reduction in smoking prevalence 3–6 months before the law (which may have been influenced by the media coverage preceding the ban), but no significant change 9 months after the law [57]. In Ireland, two studies (reported in the same publication [58]) found a nonsignificantly lower smoking prevalence 1 year after implementation of a complete smoking ban among bartenders and the general public. Other studies conducted in Spain [59], Scotland [41, 60], England [61, 62], Germany [63], and The Netherlands (a partial smoking ban exempting the hospitality industry) [64] found no significant impact of a smoking ban on smoking prevalence. Wakefield et al. found no significant impact of an incremental increase in the population covered by smoke-free restaurant-specific laws on monthly smoking prevalence in Australia [27]. However, another study conducted in Australia among youths 12–17 years old found a lower smoking prevalence with stronger smoking bans (adjusted odds ratio, 0.93; 95% confidence interval (CI), 0.92–0.94) [16]. Two US studies evaluated the effects of venue-specific smoking bans among workers most affected by those laws [65, 66]. Both studies found a decreased smoking prevalence among bartenders after smoking bans in bars, but no change in other workers [66]. Another study conducted in the US-categorized state smoking bans by the number and type of restrictions and reported their results stratified by age group [33]. State smoking bans were largely insignificant, but this is probably due to the small number of changes in state smoking bans during the period of their analysis.

### 3.4. Banning Advertising and Sponsorship of Tobacco Products

We found insufficient evidence to estimate the impact of implementation of advertising bans or restrictions. We did not identify any studies measuring smoking initiation or cessation as the outcome. Five studies examined prevalence (three among youths and two among adults), comparing rates of smoking before and after implementing advertising bans or restrictions (Table 5). Two of the youth studies showed declines in smoking prevalence; however, inferences regarding the independent effect of advertising bans were limited by the lack of a control group and long time frame between baseline and followup [67, 68]. The other youth study, conducted in Australia, showed an increased smoking prevalence with stronger point-of-purchase and outdoor advertising bans, after adjusting for demographics and other tobacco control policies (adjusted odds ratio: 1.03, 95% CI: 1.01; 1.05) [16].

Other factors influencing findings included the comprehensiveness of the ban, the level of enforcement, and industry response of shifting to indirect means of marketing. One study evaluated price and smoking prevalence in the five largest capital cities in Australia, while adjusting for a tobacco sponsorship ban that “brought two remaining states into line with the three states that had already banned tobacco sponsorship.” The authors found no association between the incremental increase in coverage of the ban and prevalence, but noted that after the ban, tobacco companies shifted resources to other outlets (e.g., point of sale) [30]. One US study found that the presence of any advertising restriction at the state level was associated with a nonstatistically significant reduction in smoking prevalence [33].

### 3.5. Health Warning Labels

We found insufficient evidence to quantify the direct impact of health warning labels on smoking prevalence. No studies examined smoking initiation. Only four studies measured smoking prevalence or cessation, and they were typically not the primary endpoints under study (Table 6).

The limited number of studies is likely due to the fact that health warning labels are implemented at the country-level, and there have been only a limited number of countries introducing new or modified warning labels. In Australia, increasing the text size from 15% to 25% of pack area was associated with a quit rate of 11%, but without a control group it is not possible to determine the net impact [69]. In addition to study design, heterogeneity could be expected as a result of differences in size, content, and design (e.g., text versus pictorial). Borland et al., using data from the International Tobacco Control Policy project, studied the effects of warning labels across four countries over four waves of data collection. Over this time period, the health warning labels on cigarette packs changed in UK (increasing text size and banning misleading product descriptors) and Australia (adding graphic images). However, the timing of these changes relative to data collection did not allow for direct comparisons of cessation behavior before and after implementation [70].

Two other studies evaluated the effects of health warning labels on smoking prevalence [30, 71]. One study reported on the effects of the introduction of 6 rotating text warnings in Australia [30], while the other reported on rotating pictorial health warning labels that covered 50% of the package in Canada [71]. Neither study reported a significant decrease in smoking prevalence.

### 3.6. Mass Media Campaigns

We found moderate strength of evidence to quantify the independent impact of mass media campaigns. Five, eight, and eight studies examined the independent effects of a mass media campaign on initiation, cessation, and prevalence, respectively (Table 7). The findings for youths were more consistent than adults, with most studies reporting a reduction of 20% to 40% in the odds of smoking initiation [72–75].

In addition to study design, key sources of heterogeneity include differences in content, tone, channels, and reach of campaigns. For example, the two studies which examined a broad campaign focused on cardiovascular disease failed to find consistent evidence of impacts on smoking prevalence [76, 77]. Among US youths, large-scale campaigns focused on tobacco industry manipulation and deception were shown to be effective at reducing initiation [75, 78, 79]. Smaller studies with other types of content were also shown to be effective [72–74]. Less consistent evidence is available for smoking cessation among youths and young adults [74, 80, 81]. Two studies evaluated campaigns that targeted ethnic groups. One, which targeted Spanish-speaking smokers, reported an increased 6-month abstinence rate among those who called into the quit line [82]. The other targeted youths of diverse racial and ethnic backgrounds, but did not report a significant effect on smoking prevalence [83]. Among adults, a mass media campaign focused on hard-hitting, graphic messages with sustained, and high levels of exposure was shown to effectively reduce smoking prevalence. A time series analysis of a mass media campaign in Australia found that an increase in 1,000 gross rating points (a measure of advertising reach and frequency) led to a reduction in adult smoking prevalence of 0.8% within 2 months, after controlling for price [27]. The study also found that the effects dissipated rapidly, suggesting that sustained high levels of exposure are necessary to maximize reductions in smoking prevalence.

## 4. Discussion

The purpose of this paper was to examine and quantify the independent impact of tobacco control policies on smoking behavior, as measured by initiation, cessation, or prevalence. Although tobacco control policies are often implemented in combination, we focused on studies that attempted to separate out the independent impact of each policy to better inform models for predicting smoking patterns. We also focused on studies that measured smoking behavior before and after policy implementation, to ensure that the proper temporal relationship was met.

### 4.1. Increasing Taxes

We found evidence that increases in tobacco pricing independently reduced smoking prevalence among youths and adults. More limited data were available for low- and middle-income countries, with some studies finding an association with decreased smoking prevalence [29, 36] and others finding no difference [29, 39, 84]. Another review found that low- and middle-income countries tended to be more price sensitive than high-income countries [85]. Based on tobacco consumption data (from estimates of cigarette sales), they estimated a price elasticity of demand of −0.8 for low- and middle-income countries versus −0.4 for high-income countries. Many factors contribute to the heterogeneity in findings, including cigarette affordability, product substitution due to wide price ranges, industry activity to reduce price for consumers, opportunities for tax avoidance, smuggling, and smokers' level of addiction.

### 4.2. Banning Smoking in Public Places

We found evidence that smoking bans can have an impact on prevalence in the general population, with greater reductions found in smaller geographic areas with limited previous legislation, compared with studies conducted at the national level. Smoking bans likely impact general population behaviour through reducing smoking opportunities and denormalizing smoking [86]. The timing of a smoking ban relative to the underlying tobacco control environment may influence its effectiveness. For example, in settings with limited tobacco control activities, the implementation of a comprehensive ban may trigger a greater shift in social norms. In other settings, implementation may represent an incremental change in the coverage of smoke-free places after years of social norm change and prevalence declines. Different impacts on smoking behaviour would be expected under these scenarios. The effectiveness of a smoking ban also depends on the strength of prior legislation, comprehensiveness of legislation, level of enforcement, and public support [87]. Public support tends to be high and increases after implementation [86].

The International Agency for Research on Cancer (IARC) found sufficient or strong evidence that smoke-free workplaces reduce cigarette consumption and increase cessation rates and that smoke-free policies reduce youth tobacco use [86]. The authors also concluded that a greater decline in smoking could be expected when the policy was part of a comprehensive tobacco control program. In the present paper, we excluded studies that examined specific workplace policies on employee behavior, in order to estimate impacts across the entire population. The studies in the IARC review were all conducted in high-income countries. With the increased adoption of smoking bans in low- and middle-income countries, more evaluation is needed.

### 4.3. Banning Advertising and Sponsorship of Tobacco Products

We found insufficient evidence to estimate the direct impact of advertising bans or restrictions on smoking initiation, cessation, or prevalence in the general population. The youth studies suggest that advertising bans may play a role in reducing smoking; however, methodological limitations restrict inferences that can be drawn.

Despite limited direct evidence of the impact of advertising bans, the role of tobacco advertising on smoking initiation is well established [88–91]. Advertising increases positive user imagery of tobacco, distorts the utility of tobacco use, increases curiosity about tobacco use [91], and influences normative beliefs and perceptions of tobacco use prevalence [92], all predictive of future smoking experimentation. Youth exposure to tobacco marketing has been associated with a doubling of the chances of initiation [93]. Comprehensive bans are the only effective way to eliminate tobacco marketing exposure, as the tobacco industry subverts restrictions by substituting marketing channels are not covered by existing laws [94].

### 4.4. Health Warning Labels

We found insufficient evidence describing the direct impact of introducing or strengthening cigarette warning labels on smoking initiation, cessation, or prevalence. The few studies that were identified were not designed specifically to address the impact of warning labels on these outcomes.

Cigarette health warning labels are a means for delivering messages about health risks from smoking and resources for obtaining help to quit. Warning labels can be implemented with little cost to governments, in comparison with mass media campaigns [95, 96]. Despite the limited direct evidence, indirect evidence describes the impact of warning messages on knowledge, salience, and cognitive processing (reading, thinking about, and discussing the warning labels) and the association between these intermediate outcomes and quit intentions, quit attempts, or cessation behavior [97]. Health warnings increase knowledge of health effects [95, 98] and have been cited as a motivating factor among quitters [99]. Studies evaluating graphic, pictorial warning labels in Canada and Australia have shown high levels of cognitive processing [96, 98, 100] and an association between cognitive processing and quitting intention and behavior [70, 98, 100, 101]. In Malaysia, a country with small, text-based warnings, a cross-sectional association was observed between cognitive processing of warning labels and intention to quit and self-efficacy among male smokers [102]. These studies provide indirect evidence for a role of health warning labels in smoking behavior.

### 4.5. Mass Media Campaigns

We found evidence that mass media campaigns can have an independent effect on reducing initiation of smoking in youths and prevalence in adults [73–75]. Differences observed in the impact of mass media campaigns are likely due, in part, to differences in content, tone, and reach. Although it is not clear which types of messages work best, behavioral research has suggested that adult audiences are most likely to respond to graphic depictions of the health consequences of smoking, and that youth audiences are more likely to respond to messages about tobacco industry deception and manipulation [103–105]. Conversely, messages focusing on smoking as an adult choice, commonly used in tobacco industry sponsored campaigns, have been shown to be ineffective or even increase youth tobacco use [103, 104, 106]. Campaign messages need to be sufficiently funded to ensure enough exposure [103, 104], tailored to the audience, and varied and rotated to keep them salient [88, 104, 105].

Our findings are consistent with prior evidence. A recent National Cancer Institute monograph concluded that mass media campaigns, even those independent of other community-wide programs, are effective at reducing smoking prevalence [103]. Several reviews have concluded that mass media campaigns are effective in reducing youth tobacco use, specifically when combined with other tobacco control programs [104, 107]. A Cochrane review, however, concluded that tobacco control programs with mass media components can be effective in reducing adult smoking, but the evidence is based on studies of “variable quality” and the “specific contribution of the mass media component is unclear” [108].

### 4.6. Limitations

Our paper had several limitations. First, we only included studies that evaluated the independent impact of a policy or intervention, thereby excluding studies of multicomponent tobacco control programs. Many studies have demonstrated the effectiveness of multicomponent tobacco control programs [109–111]. Policies are most often implemented in combination with others. Even if they are not implemented on the same date, it is often not possible to analytically separate out their independent contributions. However, evaluation of multicomponent interventions inherently captures the potential synergistic or duplicative effects of policies implemented in combination and provides a range of achievable impacts at the population level.

By limiting our paper to the effects of tobacco control interventions on smoking prevalence, initiation, and cessation, we excluded several other intermediate outcomes, such as tobacco consumption. Tobacco consumption data (i.e., cigarette sales data) is routinely collected in many countries, whereas prevalence data requires conducting surveys. Many studies have demonstrated that increased tobacco prices lead to lower per capita cigarette consumption in low-, medium-, and high-income countries [94, 112–142]. Additionally, studies evaluating per capita consumption have generally found an association between comprehensive advertising bans and reduced cigarette consumption in both developed and developing countries [94, 126]. Including tobacco consumption, data could have strengthened our conclusions on the effectiveness of these interventions. However, tobacco consumption data does not allow us to distinguish between reduced smoking prevalence and reduced consumption among smokers. Policies and interventions can affect outcomes beyond smoking behavior [143]. As mentioned earlier, health warning labels can impact on knowledge, salience, and cognitive processing, which can influence behavior. Inclusion of these other outcomes could have strengthened our results.

Many tobacco control interventions affect entire communities or countries. Complex social and cultural contexts often limit the ability to identify comparable groups of individuals or regions of study. As a result, comparison groups may vary on characteristics related to smoking behavior in the population [103]. In the absence of comparable control groups, time series or pre-/post- studies provide useful evidence for effectiveness. Information on prior trends is preferred to a single estimate before and after an intervention [103], but this requires rich surveillance data which may not be available in all settings. In longitudinal studies, participant attrition leads to the potential for selection bias and a reduction in statistical power.

Most studies included in this paper were from high-income countries, in part because they are more likely to have implemented policies. However, they may not necessarily predict the impact in low- and middle-income countries. With global expansion of tobacco control efforts through the FCTC, a wide range of programs and policies are being implemented across the world. Rigorous evaluation of these programs is needed to determine the effectiveness in reducing tobacco use. Previous studies have suggested that lower income populations may be more sensitive to demand-side tobacco control activities. For example, it is well established that low-income populations are more sensitive to changes in price [85]. In addition, Blecher found a greater association between strength of advertising bans and per capita cigarette consumption in developing compared with developed countries [126]. The author suggested that the lower level of awareness of tobacco-related harm increases the public's susceptibility to tobacco marketing. Similarly, introduction of health warning labels may have a greater impact in settings with fewer other sources of antitobacco information. In addition, implementation of smoking bans could produce a greater change in social norms than in settings, where smoking has been declining for years due to concerted tobacco control efforts.

## 5. Conclusion/Recommendations

Estimates of the impact of tobacco control policies are critical for setting achievable targets for reductions in smoking prevalence. For several of the policies, we found high or moderately strong evidence that these interventions can independently reduce smoking prevalence in the general population. However, a wide range of impacts were observed. Factors influencing the observed impact likely include the strength of the policy and level of enforcement; promotion around its implementation; the content, tone, and reach of a mass media campaign; the underlying tobacco control environment; strategic activities of the tobacco industry to dampen the effect of policies and programs. Future studies should attempt to characterize these factors to understand the variation in impacts.

Simulation models should account for this uncertainty by incorporating sensitivity analyses or probabilistic approaches to evaluate a possible range of effectiveness. For some policies, indirect evidence can be incorporated with simplifying assumptions, such as studies using per capita consumption or shorter-term outcomes that have been shown to predict subsequent smoking behavior change. Finally, given the number of studies evaluating comprehensive, multicomponent programs, models could be developed to incorporate this evidence, rather than assuming that individual interventions implemented in combination will act independently. Any approach to predict future smoking patterns will require some simplifying assumptions, but modeling can provide critical tools to inform decision-making and priority setting and to set realistic goals for reducing smoking prevalence and improving public health.

## Figures and Tables

**Table 1:** Definitions of the tobacco control interventions.

Key question	Intervention definition	Study design criteria
Taxation	Any change in price or tax on cigarettes	
Banning smoking in public places	Policy or legislative change at the national, state, or community level that prohibits or restricts smoking in indoor environments. The target of the ban or restriction could include worksites, public places, and bars and/or restaurants. Smoking bans are classified as (1) complete when 100% smoke-free or no smoking allowed in any indoor area; (2) partial when smoking is restricted or limited to designated areas. We excluded smoking bans that were conducted among a specialized population, such as hospitalized patients, military recruits, or prisoners. While we did not include specific worksite smoking bans, we included studies conducted among specific workers if it evaluated a policy or legislative smoking ban	(i) cluster randomized trial (ii) longitudinal study (iii) pre-/post- repeated cross-sectional study with a comparison group (iv) pre-/post- repeated cross-sectional study without a comparison group* (v) time series analysis

Banning advertising and sponsorship	Ban or restriction on advertising or sponsorship, which may include television, radio, print, or internet advertising, point of purchase displays, product placement, and sponsorship of any type of event	

Health warning labels	Any required changes to the packaging of tobacco products intended to disseminate health warnings or eliminate the use of terms implying a safer product (e.g., changes to graphic images or text of health warning labels or restrictions on the use of terms, such as “mild,” “low tar,” or “light”)	

Mass media campaigns	Any campaign intended to reduce tobacco use using “channels of communication such as television, radio, newspapers, billboards, posters, leaflets, or booklets intended to reach large numbers of people, which are not dependent on person-to-person contact” [108]	

*Excluded from the mass media campaign review.

**Table 2:** Overall summary of the impact of tobacco control interventions on smoking initiation, cessation, and prevalence.

Intervention	Smoking behavior
Increasing the price through taxation	*Overall*: high* evidence to estimate the independent impact on smoking behavior Initiation: moderate evidence, 4 out of 5 longitudinal studies demonstrated some effectiveness; PPE of initiation ranged from −0.65 to −0.09 Cessation: moderate evidence, price elasticity of cessation ranged from 0.375 to 1.17 Prevalence: high evidence, suggesting effectiveness PPEs ranged from −1.41 to −0.10 among youths and −0.45 to 0.10 among adults

Banning smoking in public places	*Overall*: moderate evidence to estimate the independent impact on smoking behavior Initiation: low evidence, unable to make a conclusion due to equivocal results Cessation: low evidence, 2 of 3 longitudinal studies with comparison groups did not find a significant change in cessation rates after implementation Prevalence: moderate evidence, suggesting effectiveness; Percentage change in prevalence^†^ ranged from −31.9% to −7.4% compared with control groups after 1 to 3.5 years

Banning advertising and sponsorship of tobacco products	*Overall*: insufficient evidence to estimate the independent impact on smoking behavior Initiation: insufficient evidence, unable to make a conclusion because no studies were included Cessation: insufficient evidence, unable to make a conclusion because no studies were included Prevalence: low evidence, unable to make a conclusion due to low quality studies; Two studies among adults showing no effectiveness, 2 studies among youths showing some effectiveness^‡^, and 1 found an increased prevalence with stronger laws

Educating people about the dangers of smoking through health warning labels	*Overall*: insufficient evidence to estimate the independent impact on smoking behavior Initiation: insufficient evidence, unable to make a conclusion because no studies were included Cessation: low evidence, 2 studies showing no effectiveness Prevalence: low evidence, 2 studies showing no effectiveness

Educating people about the dangers of smoking through mass media campaigns	*Overall*: moderate evidence to estimate the independent impact on smoking behavior Initiation: moderate evidence, suggesting effectiveness One cluster RCT demonstrated no effectiveness, but 4 longitudinal studies suggested a reduced initiation rate (odds of initiating smoking ranged from 0.67 to 0.8)^¶^ Cessation: low evidence, unable to make a conclusion due to equivocal results. Seven studies with comparison groups showed equivocal results^*∧*^ Prevalence: moderate evidence, suggesting effectiveness. Odds of being a smoker 1 to 6 years after start of intervention* ranged from 0.62 to 0.93^§^, but one cluster RCT showed no effect on smoking prevalence

*Grading classification: *high* strength of evidence indicates high confidence that the evidence reflects the true effect, and further research is very unlikely to change the result. *Moderate* strength of evidence indicates moderate confidence that the evidence reflects the true effect, and further research may change the result. *Low* strength of evidence indicates low confidence that the evidence reflects the true effect, and further research is likely to change the result. *Insufficient* indicates that no evidence was available.

^†^One of these studies stratified results by gender and age (% impact on prevalence rate after 2 years for those under age 45 years = −7.4% and for those aged 45 years and older = −1.4%).

^‡^These studies had severe methodological flaws that limit our ability to make conclusions.

^¶^The strongest study methodologically showed a hazard ratio of 0.8 (95% CI: 0.71, 0.91; *P* = 0.001) per 10,000 GRP cumulative exposure.

^*∧*^Two of the pre-/post- cross-sectional studies were methodologically stronger than the others. One study reported an odds ratio of cessation = 1.27 (95% CI: 0.77 to 2.08). The other reported a relative risk of quitting = 1.1 (95% CI: 0.98 to 1.24) per 5,000 GRPS.

^§^Additionally, a well-conducted time series analysis reported a decrease in percentage point prevalence two months later of −0.00077 per 1 GRP per month increase (*P* = 0.025). This is the equivalent of each person viewing an average of 4 ads per month to achieve a 0.30 percentage point decline in smoking prevalence.

CI: confidence intervals; GRP: gross rating point; PPE: price participation elasticity; RCT: randomized controlled trial.

**Table 3:** Effects of taxation/price on smoking initiation, cessation, and prevalence.

Author, year	Country (data source)	Study design	Dates of data collection	Population (*n*)	Intervention (currency)	Smoking measure	Effect on smoking initiation, cessation, or prevalence
Smoking initiation							

Nonnemaker and Farrelly, 2011 [7]	US (NLSY97)	Longitudinal	1997–2006	Youths, age 12–17 (8984); mean age = 15 51% male	Change in real state-level taxes* (1996 US$)	Ever smoked a cigarette	*Overall * OR (se): 0.88 (0.06), *P* = 0.06 Elasticity: −0.09 *Males * OR (se): 0.93 (0.08), *P* = 0.41 Elasticity: −0.05 *Females * OR (se): 0.81 (0.06), *P* = 0.001 Elasticity: −0.15

Sen and Wirjanto, 2010 [8]	Canada (Waterloo Smoking Prevention Project)	Longitudinal	1993–1996	Youths, grade 9 (2364)	Change in real excise and sales taxes (C$)	Smoked in past month	Elasticity: −0.5, *P* < 0.1

Tauras, 2005 [10]	US (MTF)	Longitudinal study	1976–1995	Youths, high school seniors (5,383)	Changes in real price* (1982–1984 US$)	Progression from nondaily to daily smoking	Coeff. (*z*-statistic): −0.46 (−2.27), *P* < 0.05 Elasticity: −0.65

Cawley et al., 2004 [9]	US (NLSY97)	Longitudinal study	1997–2000	Youths, ages 12–16 (12,282)	Changes in real price* (NR)	Smoking any positive quantity of cigarettes	Coeff. (*z*-statistic): −0.15 (−1.45), *P* < 0.1 *Males * coeff. (*z*-statistic): −0.28 (−2.03), *P* < 0.05 *Females * coeff. (*z*-statistic): −0.03 (−0.17), *P* > 0.05

DeCicca et al., 2002 [11]	US (NELS:88)	Longitudinal study	1988–1992	Youths, 8th grade (12,089)	Changes in nominal tax* (1988 US$)	Daily smoking	Coeff. (*t* value): −0.003 (−1.31), *P* > 0.05

Smoking cessation							

Ross et al., 2010 [22]	US and Canada (ITC)	Longitudinal	2002–2004	Adults (1990): mean age = 41 41% male	(1) change in real price (US$); (2) change in province-level cigarette tax (US$)	Quit smoking	(1) coeff. (se): 0.0064 (0.0038), *P* < 0.1 (2) coeff. (se): 0.0036 (0.0046)

Saenz-de-Miera et al., 2010 [23]	Mexico (ITC-Mexico)	Longitudinal	2006-2007	Adults, age 18+ (728): mean age = 39 61% male	SPST tax increased from 110% of price to retailers to 140% in 2007	Quit smoking for at least 30 days	Quit rate: 13.1% (95% CI, 9.7 to 16.5%)

Hanewinkel and Isensee, 2007 [24]	Germany (NA)	Longitudinal study	01/2002–09/2005	Adults, age 14+; mean age 46.5	Before and after each of 5 tax increases (Euros)	Quit rates 1–4 months after tax increase	Quit rates ranged from 4% to 7.9%; OR = 1.58, *P* < 0.05

Tauras and Chaloupka, 1999 [12]	US (MTF)	Longitudinal study	NR	Youths, high school seniors	Changes in real price* (1982–1984 US$)	30-day abstinence	*Males * Coeff.: 0.746; *P* < 0.05; Price elasticity: 1.15; *Females * Coeff.: 0.742; *P* < 0.01; Elasticity: 1.17

Franz, 2008 [25]	US (BRFSS)	Before/after w/o comparison	1993–2000	Adults, age 18+	Changes in real price* (1995 US$)	Quit daily smoking within previous year	Baseline: 13.8%; Final: 14.3%; Coeff.: 0.023, *P* < 0.001 Elasticity: 0.375

Reed et al., 2008 [26]	US (CTS)	Before/after w/o comparison	1995–1999	Adults, age 25+	Before and after Proposition 10 and MSA, which raised price by US$ 0.95 (NA)	Quit smoking entirely within previous year	OR = 1.04; *P* = 0.76

Smoking prevalence among youth							

Grossman, 2005 [13]	US (MTF)	Time series	1975–2003	Youths, high school seniors	Changes in real price* (1975 US$)	Smoked in past 30 days	Coeff. (*t*-statistic): −0.12 (−5.23); *P* < 0.05

Tauras and Chaloupka, 1999 [14]	US (MTF)	Longitudinal study	1976–1993	Youths, high school seniors	Changes in real price* (1982–1984 US$)	Smoked in past 30 days	Coeff (*t*-ratio): −0.03 (−2.38); *P* < 0.05 Elasticity: −0.10

Kostova et al., 2011 [15]	17 LMIC (GYTS)	Before/after w/comparison	1999–2006	Youths, age 13–15	Change in real price (2000 US$)	Smoked in the past month	Elasticity for local brands: −0.74 Elasticity for foreign brands: −1.09

White et al., 2011 [16]	Australia (cross-sectional surveys of secondary schools)	Before/after w/comparison	1990–2005	Youths, age 12-17	Change in state-specific cigarette prices (2005 AU$)	Smoked in the past month	aOR = 0.98 (95% CI: 0.97; 0.99) (1 cent increase in change in price per stick)

Carpenter and Cook, 2008 [17]	US (YRBS)	Before/after w/o comparison	1991–2005	Youths, grades 9–12	Changes in real price* (2005 US$)	Smoked in past 30 days	Coeff. (se): −0.286 (0.101); *P* < 0.01 Elasticity: −0.56

Ding, 2003 [18]	US (MTF)	Before/after w/o comparison	1976–1998	Youths, high school seniors	Changes in real price* (US$)	Smoked in past 30 days	Elasticity (se): −1.41 (0.83); *P* = 0.10 *Males * Elasticity (se): 0.29 (1.03), *P* = 0.78 *Females * elasticity (se): −2.98 (0.69); *P* < 0.05

Waller et al., 2003 [19]	Canada (OSDUS)	Before/after w/o comparison	1977–2001	Youths, grades 7, 9, 11, and 13	Before and after a decrease of C$10 in taxes (C$)	Smoked > 1 cigarette in past 12 months	Overall results for smoking prevalence showed a significant discontinuity effect with a negative slope until 1993 and upward jump at the discontinuity point and leveling off after 1993

Gruber, 2000 [20]	US (MTF)	Before/after w/o comparison	1991–1997	Youths, grades 8, 10 and 12	Changes in real price* (1982 US$)	Smoked in past 30 days	Coeff. (se): −0.955 (0.034); *P* > 0.05 Elasticity: −0.31 *8th and 10th graders * Coeff. (se): −0.03 (0.035); *P* > 0.05 Elasticity: −0.21 *12th graders * Coeff. (se): −0.148 (0.078); *P* < 0.05 Elasticity: −0.67

Chaloupka and Pacula, 1998 [21]	US (MTF)	Before/after w/o comparison	1975–1994	Youths, grades 8, 10 and 12; mean age = 16.3	Changes in real price* (1982–1984 US$)	Smoked in past 30 days	Coeff. (*t*-ratio): −0.004 (−2.62); *P* < 0.05 Elasticity: −0.62

Smoking prevalence among adults							

Wakefield et al., 2008 [27]	Australia (Roy Morgan Single Source)	Time series	1995–2006	Adults, age 18+	Cigarette costliness^‡^ (NR)	Smoke factory-made cigarettes	Coeff. (se): −8.802 (2.891); *P* < 0.003

Azagba and Sharaf, 2011 [28]	Canada (National Population Health Survey)	Longitudinal	1999–2009	Adults, ages 12–65 (56,770) mean age = 38 50% male	Changes in real tax (2000 C$)	Daily and occasional smokers	Elasticity: −0.23 *Males * elasticity: −0.32; *P* < 0.01 *Females * elasticity: −0.12; *P* > 0.1

Lance et al., 2004 [29]	China (CHNS); Russia (RLMS)	Longitudinal study	China: 1993–1997; Russia: 1996–2000	Adults, age 13+; 100% male	Change in nominal price (China: yuan; Russia: ruble)	NR	*China * coeff. (se): −0.123 (0.165); *P* > 0.05 Elasticity = −0.045 *Russia * coeff. (se): −0.011 (0.003); *P* < 0.01 Elasticity = −0.101

Bogdanovica et al., 2011 [38]	European Union (Euro-barometer Surveys)	Before/after w/o comparison	2006–2009	Adults, age 15+	Change in cigarette affordability	Smoking prevalence	Correlation: −0.06; *P* = 0.77

Siahpush et al., 2009 [30]	Australia (Roy Morgan Single Source)	Before/after w/o comparison	1991–2006	Adults, age 18+; ages 18–29: 21%; ages 30–49: 41%; ages 50+: 38%; 48% male	Changes in real price^‡^ (2006 AU$)	Do you now smoke factory-made cigarettes? In the last month, have you smoked any roll-your-own cigarettes?	aRR (95% CI) = 0.974 (0.951 to 0.997) *Price × income * Price × medium income: 1.024 (1.015 to 1.023) Price × high income: 1.025 (1.016 to 1.035)

Gospodinov and Irvine, 2009 [144]	Canada (CTUMS)	Before/after w/o comparison	2000–2005	Adults, age 20+	Changes in real price, based on Canadian Socioeconomic Information Management system (2001 C$)	Occasional or daily smoker	Coeff.: −0.0008 (se = 0.0006); *P* > 0.05 Elasticity: −0.299 (se = 0.224) (95% CI: 0.133–−0.760)

DeCicca and McLeod, 2008 [31]	US (BRFSS)	Before/after w/o comparison	2000–2005	Adults, aged 45–65	Several state cigarette tax increases* (2001 US$)	Daily smoker	*Daily smoking * Coeff.: −0.0098 (se = 0.0036); *P* < 0.05 Elasticity: −0.21: *smoked on some days * Coeff.: −0.0110 (se = 0.0032); *P* < 0.05 Elasticity: −0.20

Jimenez-Ruiz et al., 2008 [39]	Mexico (ENIGH)	Before/after w/o comparison	1994–2005	Adults, age 15+	Until 1999, 40% for filter and 15% for unfiltered; in 2005, 45.5% for both filtered and unfiltered (NR)	Household spent money on cigarettes	Coeff. (*t*-statistic): −0.0019 (1.77); *P* < 0.10 Elasticity = −0.06

Franz, 2008 [25]	US (BRFSS)	Before/after w/o comparison	1993–2000	Adults, age 18+	Changes in real price* (1995 US$)	Current smoker and smoked more than 100 cigarettes	Baseline: 22.2% Final: 17.9% Coeff.: −0.016; *P* < 0.001 Elasticity = −0.193

Franks et al., 2007 [32]	US (BRFSS)	Before/after w/o comparison	1984–2004	Adults, age 18+	Changes in real price* (2004 US$)	Current smoker	*1984–1996, lowest income quartile * Elasticity: −0.45 (−0.67–−0.22); *P* < 0.01 *1984–1996, other income quartiles * Elasticity: −0.22 (−0.35–−0.10), *P* < 0.01 *1997*–*2004, lowest income quartile * Elasticity: −0.14 (−0.36–0.08) * 1997*–*2004*, *other income quartiles * Elasticity: −0.07 (−0.18–0.05)

Sloan and Trogdon, 2004 [33]	US (BRFSS)	Before/after w/o comparison	1990–2002	Adults, age 18+; 35–46% male	Changes in real price* (2002 US$)	Daily or some day smoker	*18 to 20 years old * Coeff. (se): −0.025 (0.012); *P* < 0.05 *21 to 24 years old * Coeff. (se): −0.011 (0.008); *P* > 0.05 *25 to 44 years old * Coeff. (se): −0.009 (0.005); *P* < 0.05 *45 to 64 years old * Coeff. (se): −0.008 (0.007); *P* > 0.05 *65+ years old * Coeff. (se): −0.010 (0.004); *P* < 0.05

Gallus et al., 2003 [34]	Italy	Before/after w/o comparison	1970–2000	Adults, age 15+	Changes in real price, taxes represented 74.7% of cost in 2000 (NR)	NR	Elasticity (se): −0.30 (0.05); *P* < 0.001

Scollo et al., 2003 [35]	Australia (NTC)	Before/after w/o comparison	1997–2000	Adults, ages 18–40	Multiple changes to the taxation structure, including the end of the State franchise fees in Aug 97, a change from a weight to a stick-based system of levying excise duty in Nov 99, and the imposition of the Goods and Services Tax in Jul 00^‡^ (NR)	NR	Prevalence (May 1997): 29.5% Prevalence (Nov 1998): 27.9% Prevalence (Nov 2000): 26.7% Change percentage from May 1997 to Nov 1998: −5.42% Change percentage from Nov 1998 to Nov 2000: −4.30%

Arunatilake, 2002 [84]	Sri Lanka	Before/after w/o comparison	1991–2000	Age < 20: 40%; age 20–30: 18%; age 30–40: 13%; age 40–50: 12%; age 50–60: 9%; age 60+: 8%; 100% male	Annual increases in tax, ranging from 27.6% of selling price in 1995 to 76.8% in 2000 (NR)	NR	Elasticity: 0.10, *P* < 0.1

van Walbeek, 2002 [36]	South Africa (AMPS)	Before/after w/o comparison	1993–2000	Adults, age 16+; ages 16–24: 28%; ages 25–34: 26%; ages 35–49: 26%; ages 50+: 21%; 48% male	Increases in the real price of cigarettes by 93% (1995 Rand)	Smoking prevalence is defined as the number of respondents who declare cigarette usage expressed as a percentage of the population	1993 Prevalence: 32.6% 2000 Prevalence: 27.1% Change percentage: −16.9% Elasticity: −0.30

Farrelly et al., 2001 [37]	US (NHIS)	Before/after w/o comparison	1976–1993	Adults, age 18+; mean age 43.9; 47% male	Changes in the real price* (1982–1984 US$)	Smoked at least 100 cigarettes during lifetime and currently smoke cigarettes every day or some days	Elasticity: −0.13 *Males * elasticity: −0.03 *Females * elasticity: −0.19

*Data obtained from the tax burden on tobacco.

^‡^Data was obtained from the Australian Retail Tobacconist.

Unless otherwise specified, elasticity is price participation elasticity (PPE, percentage change in smoking prevalence for one percentage change in price).

All odds ratios and relative risks can be interpreted as the change in outcome comparing the intervention to control group or after versus before an intervention or a unit increase in the intervention (e.g., 1$ in tax increase).

AMPS: All Media and Products Survey; aOR: adjusted odds ratio; AU$: Australian dollars; BRFSS: Centers for Disease Control and Prevention's Behavioral Risk Factor Surveillance System; C$: Canadian dollars; CHNS: China Health and Nutrition Survey; CI: confidence interval; Coeff.: coefficient; CTS: California Tobacco Survey; CTUMS: Statistics Canada/Health Canada Canadian Tobacco Use Monitoring Survey; GYTS: Global Youth Tobacco Survey; ENIGH: National Household Income and Expenditure Survey; ITC: International Tobacco Control Policy Evaluation Survey; LMIC: low- and middle-income countries; MS: Master Settlement Agreement; MTF: Monitoring the Future: a Continuing Study of American Youth; NA: not applicable; NELS: 88: National Education Longitudinal Survey of 1988; NHIS: National Health Interview Surveys; NLSY97: National Longitudinal Survey of Youth 1997 Cohort; NR: not reported; NTC: National Tobacco Campaign Evaluation respondent surveys; OR: odds ratio; OSDUS: Ontario Student Drug Use Survey; RLMS: Russian Longitudinal Monitoring Survey; se: standard error; SPST: special production and services tax; US: United States; US$: United States dollars; YRBS: Youth Risk Behavior Survey.

**Table 4:** Effects of banning smoking in public places on smoking initiation, cessation, and prevalence.

Author, year	Country (data source)	Study design	Dates of data collection	Population	Intervention, *n *	Smoking measure	Effect on smoking initiation, cessation, or prevalence
Smoking initiation							

Hawkins et al., 2011 [41]	England, Scotland (MCS)	Longitudinal	2000–2007	Adults mean age = 29	(I) complete ban, including restaurants and/or bars, 1072 (f) and 632 (m) (C) no smoking ban, 4158 (f) and 2624 (m)	Daily smoking	Initiation rates at followup, females: (I) 6.2% (C) 7.3% aOR = 0.75 (95% CI: 0.58; 0.97) Initiation rates at followup, males: (I) 3.6% (C) 4.5% aOR = 0.81 (95% CI: 0.48; 1.37)

Klein, 2008 [42]	US (MACC)	Longitudinal	2000–2006	Youths, age 12–16	(I) complete ban in restaurants and/or bars (C) smoking areas designated or not restricted	Ever smoked at least a whole cigarette	aOR = 1.08 (95% CI: 1.00;, 1.16)

Smoking cessation							

Hawkins et al., 2011 [41]	England, Scotland (MCS)	Longitudinal	2000–2007	Adults Mean age = 29	(I) complete ban, including restaurants and/or bars, 1072 (f) and 632 (m) (C) no smoking ban, 4158 (f) and 2624 (m)	Not smoking any cigarettes	Quit rates within 1 year after ban, females: (I) 16.0% (C) 24.0% aOR = 0.65 (95% CI: 0.47; 0.89) Quit rates within 1 year after ban, males: (I) 20.5% (C) 28.8% aOR = 0.66 (95% CI: 0.46; 0.93)

Biener et al., 2010 [43]	US (UMass Tobacco Study)	Longitudinal	2001–2006	Adults, age 18+, Age 18–30: 25% Age 31–59: 65% Age 60+: 10%–46% male	(I) change in town's workplace or restaurant smoking ban, 1162 (C) no change, 1473	3-month abstinence	Quit rates within 2 years after ban: (I) 13.1% (C) 13.8% aOR = 0.95 (95% CI: 0.69; 1.31)

Hyland et al., 2009 [44]	UK (ITC)	Longitudinal	2006-2007	Adults, age 18+	(I) complete ban, including restaurants and/or bars, in Scotland, 507 (C) other parts of UK, 828	Smoked at least once/month and smoked at least 100 cigarettes lifetime	Quit rates 1 year after intervention: (I) 19% (95% CI: 9.8; 29%) (C) 21% (95% CI: 14; 28%) aOR = 0.91 (95% CI: 0.47; 1.7)

De Chaisemartin et al., 2011 [50]	France (Consultation Dependance Tabagique)	Longitudinal	2004–2008	Adults	(I) complete ban in workplaces, 5963	Smoked 0 cigarettes/day and all expired CO measures <9 ppm	Mean difference in quit rates between employed and unemployed: 7.0%

Bauza-Amengual et al., 2010 [45]	Spain (original data collection)	Longitudinal	2006-2007	Adults, age 18+	(I) complete ban, including restaurants and/or bars^††^	Quit smoking (self-reported)	Quit rates 1 month after ban: 9.5% Quit rates 6 months after ban: 13.8%

Murphy et al., 2010 [46]	US (original data collection)	Longitudinal	2002–2005	Adults, age 18+, 20% male, mean age = 37 years	(I) complete ban, including restaurants and/or bars, 237	Quit smoking	Quit rate 2 years after ban: 14%

Orbell et al., 2009 [47]	England (original data collection)	Longitudinal	2007	Adults, age 18+, 57% male, Mean age = 36 years	(I) complete ban, including restaurants and/or bars, 84	Quit smoking	Quit rates 3 months after ban: 15.5%

Martinez-Sanchez et al., 2009 [48]	Spain (original data collection)	Longitudinal	2005-2006	Adults	(I) complete ban, including restaurants and/or bars^††^, 118	Daily or occasional smokers with salivary cotinine concentration ≤35 ng/mL per cigarette smoked	Quit rate 1 year after ban: 5.1%

Fowkes et al., 2008 [49]	Scotland (AAA Trial)	Longitudinal	1998–2007	Adults, age 50–75 33% male mean age = 60.9	(I) complete ban, including restaurants and/or bars, 1141	Self-reported; must have quit for at least 3 months	Change in smoking cessation pattern during 2006, with increase in quit rates (5.1%) in 3-month period prior to ban

Smoking prevalence							

Mackay et al., 2011 [57]	Scotland (Scottish Household Survey)	Time series	1999–2010	NR	(I) complete ban, including restaurants and/or bars	Current smoker	Coeff. for 3–6 mos prior to law: −1.70 (95% CI: −2.38, −1.02), *P* < 0.001 Coeff. for 9 mos after law: −0.08 (95% CI: −0.39, 0.22); *P* = 0.59

Wakefield et al., 2008 [27]	Australia (Roy Morgan Single Source)	Time series	1995–2006	Adults, age 18+	(I) complete ban, restaurants only	Smoke factory-made cigarettes	Coeff. (se): −0.0104 (0.0103); *P* = 0.317

Anger et al., 2011 [63]	Germany (SOEP)	Longitudinal	2002–2008	Adults, mean age = 47 47% male	(I) complete ban, including restaurants and/or bars	Current smoker	Coeff.: −0.004 (se: 0.008); *P* > 0.05

Hawkins et al., 2011 [41]	England; Scotland (MCS)	Longitudinal	2000–2007	Adults, mean age = 29	(I) complete ban, including restaurants and/or bars, 1522 (f) and 904 (m); (C) no smoking ban, 5954 (f) and 3757 (m)	Daily smoking	Smoking prevalence at baseline, females: (I) 31.0% (C) 29.8% Smoking prevalence at followup, females: (I) 30.3% (C) 27.7% aOR = 1.15 (95% CI: 0.95; 1.40) Smoking prevalence at baseline, males: (I) 31.5% (C) 29.5% Smoking prevalence at followup, males: (I) 27.5% (C) 24.2% aOR = 1.24 (95% CI: 0.95; 1.61)

Mullally et al., 2009 [58]	Ireland (All-Ireland Bar Study)	Longitudinal	2004-2005	Adults, age 18+ 71% male Mean age = 33	(I) complete ban, including restaurants and/or bars	Combined self report and cotinine measures	Smoking prevalence prior to law: 56.1% Smoking prevalence 1 year after law: 51.4%; *P* = 0.125

Klein et al., 2009 [54]	US (MACC)	Longitudinal	2000–2006	Youths, age 12–16 49% male	(I) complete ban in restaurants and/or bars, 1028; (C) smoking areas designated or not restricted, 3205	Smoked in the past month	aOR = 1.06 (95% CI: 0.93; 1.21)

Bitler et al., 2011 [65]	US (TUS-CPS)	Before/after w/comparison	1992–2007	Adults, age 18+	Strength of state smoking bans in bars^§^	Daily or someday smoker	OR = 0.78 (95% CI: 0.64 to 0.94)

White et al., 2011 [16]	Australia (cross-sectional surveys of secondary schools)	Before/after w/comparison	1990–2005	Youths, age 12–17	Scoring system based on the extent to which policies have been adopted	Smoked in the past month	aOR = 0.93 (95% CI: 0.92; 0.94)

Hahn et al., 2010 [51]	US	Before/after w/comparison	2004–2008	Youths, age 18–24 31–39% male	(I) complete smoking ban, including restaurants and/or bars, 897*, 469** (C) delayed smoking ban, including restaurants and/or bars, 703*, 701**	Smoked in past 30-days	Smoking prevalence (I) before ban: 28.0%; 3.5 years after ban: 19.4%; *P* = 0.005 Smoking prevalence (C) before ban: 21.5%; 8 months after ban: 16.9%; *P* = 0.03

Bitler et al., 2010 [66]	US (TUS-CPS)	Before/after w/comparison	1992–2007	Adults, age 18+	Venue-specific Impact Teen ratings	Smoked at least some days	Coeff. for private workplace SCIAL among private sector workers: 0.001 (se: 0.003); *P* > 0.05 Coeff. for government workplace SCIAL among government workers: 0.011 (se: 0.009); *P* > 0.05 Coeff. for public school SCIAL among school workers: −0.001 (se: 0.003); *P* > 0.05 Coeff. for private school SCIAL among school workers: −0.004 (se: 0.004); *P* > 0.05 Coeff. for restaurant SCIAL among all workers at eating/drinking places: 0.013 (se: 0.014); *P* > 0.05 Coeff. for bar SCIAL among bartenders: −0.058 (se: 0.021); *P* < 0.01

Hahn et al., 2008 [52]	US (BRFSS)	Before/after w/comparison	2001–2005	Adults, age 18+	(I) complete ban, including restaurants and/or bars, 579* and 281** (C) no smoke-free laws, 6560* and 2993**	Daily or some day smoker and smoked at least 100 cigarettes lifetime	Smoking prevalence 40 months prior to law: (I) 25.7% (95% CI: 21.2, 30.1%) (C) 28.4% (95% CI: 26.8, 30.0) Smoking prevalence 20 months after law: (I) 17.5% (11.8, 23.1) (C) 27.6% (25.2, 30.0) aOR = 0.84 (0.72, 0.97)

Lemstra et al., 2008 [53]	Canada (Canadian Community Health Survey)	Before/after w/comparison	2003–2005	Adults	(I) complete ban, including restaurants and/or bars, 1301* and 1244** (C1) Saskatchewan (C2) Canada	NR	Baseline smoking prevalence: (I) 24.1% (95% CI: 20.4, 27.7) (C1) 23.8 (22.6, 25.3) (C2) 22.9 (22.5, 23.3) Smoking prevalence 1 year after law: (I) 18.2% (15.7, 20.9) (C1) 23.8 (C2) 21.3 (20.8, 21.8)

Lee et al., 2011 [61]	England (Health Survey for England)	Before/after w/o comparison	2003–2008	Adults, age 18+	(I) complete ban, including restaurants and/or bars	Current smoker	aOR = 1.02 (95% CI: 0.94, 1.11)

Guerrero et al., 2011 [56]	Spain (National Health Survey for Spain)	Before/after w/o comparison	1993–2009	Adults, age 16–65	(I) complete ban, including restaurants and/or bars^††^	Smoked at least 100 cigarettes lifetime	Smoking prevalence in 1993: 36.18% Smoking prevalence in 2003: 30.97% Smoking prevalence in 2006 (<1 yr after ban): 29.50% Smoking prevalence in 2009 (3 yrs after ban): 31.47%

Verdonk-Kleinjan et al., 2011 [64]	The Netherlands (Continuous Survey of Smoking Habits)	Before/after w/o comparison	2003-2004	Adults, age 16–65	(I) complete ban in workplaces, 601	Daily smoking	Smoking prevalence prior to ban: 27.5% Smoking prevalence 1 month after ban: 25.5% OR = 0.87 (95% CI: 0.70; 1.08)

Mullally et al., 2009 [58]	Ireland (survey commissioned by the Office of Tobacco Control)	Before/after w/o comparison	2004-2005	Adults, age 18+	(I) complete ban, including restaurants and/or bars	Smoked more than 1 cigarette per week	Smoking prevalence prior to law: 28.3% Smoking prevalence 1 year after law: 24.8%; *P* = 0.055

Elton and Campbell, 2008 [62]	England (original data collection)	Before/after w/o comparison	2007	Adults, age 18+, age 18–24: 7% age 25–34: 12% age 35–44: 16% age 45–54: 18% age 55–64: 20% age 65–74: 14% age 75+: 13% 45% male	(I) complete ban, including restaurants and/or bars, 2054* and 1938**	Currently smoke	Baseline smoking prevalence: 22.4% Smoking prevalence 3 months after law: 22.6%

Haw and Gruer, 2007 [60]	Scotland (original data collection)	Before/after w/o comparison	2005–2007	Adults, age 16–74	(I) complete ban, including restaurants and/or bars, 1815* and 1834**	Self-reported	Baseline smoking prevalence: 35.6% Smoking prevalence after law: 35.1%

Galan et al., 2007 [59]	Spain	Before/after w/o comparison	2005-2006	Adults, age 18–64, Age 18–29: 26% Age 30–44: 40% Age 45–64: 33% 48% male	(I) complete ban, including restaurants and/or bars^††^, 1750* and 1252**	Self-reported	Baseline smoking prevalence: 31.7% Smoking prevalence after law: 32.7%

Gallus et al., 2006 [55]	Italy (DOXA)	Before/after w/o comparison	2004-2005	Adults, age 15+	(I) complete ban, including restaurants and/or bars^†^	NR	Baseline smoking prevalence: (I) 26.2% Smoking prevalence 3 months after laws: (I) 25.6%

Sloan and Trogdon, 2004 [33]	US (BRFSS)	Before/after w/o comparison	1990–2002	Adults, age 18+; 35–46% male	Categorical variables based on number and type of public places where smoking is banned: none, nominal, basic, moderate, and extensive^‡^, 1,762,686	Daily or some day smoker	Nominal^‡‡^: 0.011, 0.001, −0.001, −0.004, and 0.006 Basic^‡‡^: 0.032, −0.047, 0.009, 0.013, and 0.005 Moderate^‡‡^: 0.030, −0.015, 0.017, 0.015, and 0.008 Extensive^‡‡^: 0.013, −0.011, 0.004, −0.005, and −0.007

*Prelaw sample size.

**Postlaw sample size.

^†^Exceptions were made to the smoking ban for restaurants with separate and regulated smoking areas.

^††^There was a partial ban on smoking in restaurants and bars. Establishments of less than 100 square meters were able to decide whether or not to permit smoking. Establishments of more than 100 square meters could provide a separate smoking area with a separate ventilation system that was no larger than 30% of the total area of the premises.

^‡^Based on data from the State Legislated Actions on Tobacco Issues, 2002.

^‡‡^Results reported by age group: 18 to 20 years, 21 to 24 years, 25 to 44 years, 45 to 64 years, and 65 years and older.

^§^Based on data from Robert Wood Johnson's ImpacTeen database.

AAA: Aspirin for Asymptomatic Atherosclerosis; aOR: adjusted odds ratio; BRFSS: Behavioral Risk Factor Surveillance Survey; C: control; CI: confidence interval; CIA: clean indoor air; CO: carbon monoxide; f: females; I: intervention; ITC: International Tobacco Control Policy Evaluation Project; m: males; MACC: Minnesota Adolescent Community Cohort; MCS: Millennium Cohort Study; NR: not reported; ppm: parts per million; SOEP: Socio-Economic Panel Study; TUS-CPS: Tobacco Use Supplement to the Current Population Survey; UK: United Kingdom; US: United States.

**Table 5:** Effects of advertising and sponsorship of tobacco products on smoking prevalence.

Author, year	Country (Data source)	Study design	Dates of data collection	Population	Intervention, *n *	Smoking measure	Effect on smoking prevalence
Smoking prevalence							

White et al., 2011 [16]	Australia (cross-sectional surveys of secondary schools)	Before/after w/comparison	1990–2005	Youths, age 12–17	Scoring system based on the extent to which policies have been adopted	Smoked in the past month	aOR: 1.03 (95% CI: 1.01; 1.05)

Sloan and Trogdon, 2004 [33]	US (BRFSS)	Before/after w/o comparison	1990–2002	Adults, age 18+; 35–46% male	Any advertising restrictions*, 1,762,686	Daily or some day smoker	18 to 20 years old Coeff. (se): −0.016 (0.012); *P* > 0.05 21 to 24 years old Coeff. (se): −0.017 (0.010); *P* > 0.05 25 to 44 years old Coeff. (se): −0.005 (0.007); *P* > 0.05 45 to 64 years old Coeff. (se): −0.004 (0.006); *P* > 0.05 65+ year olds Coeff. (se): −0.006 (0.006); *P* > 0.05

Galduróz et al., 2007 [67]	Brazil (original data collection)	Before/after w/o comparison	1997–2004	Youths, age 11–18; 42% male	Advertising ban on the following media: billboard, print, radio, sponsorship, sporting or cultural activity, TV, 15,501^†^ and 21,172^‡^	Lifetime use of tobacco	Baseline prevalence: 32.7% Smoking prevalence 4 years after ad ban: 25.0%

Fielding et al., 2004 [68]	Hong Kong (original data collection)	Before/after w/o comparison	1990–2001	Youths, aged 8–10	Advertising ban on the following media: broadcast media (1990), billboards, print (1999), 824	Ever smoked	Baseline prevalence: 7.8% Follow-up smoking prevalence: 3.8%

Siahpush et al., 2009 [30]	Australia (Roy Morgan Single Source)	Before/after w/o comparison	1991–2006	Adults, age 18+; ages 18–29: 21%; ages 30–49: 41%; ages 50+: 38%; 48% male	National ban on tobacco sponsorship, bringing 2 remaining states into line with the 3 states that had already banned tobacco sponsorship at the state level (December, 1995), 515,866	Do you now smoke factory-made cigarettes? In the last month, have you smoked any roll-your-own cigarettes?	aRR = 1.00, *P* = 0.90

*Based on data from the Centers for Disease Control and Prevention's State Tobacco Activities Tracking and Evaluation (STATE) System.

^†^Preban sample size.

^‡^Postban sample size.

aRR: adjusted rate ratio; aOR: adjusted odds ratio; BRFSS: Behavioral Risk Factor Surveillance Survey; CI: confidence interval; coeff.: coefficient; se: standard error.

**Table 6:** Effects of health warning labels on smoking cessation and prevalence.

Author, year	Country (data source)	Study design	Dates of data collection	Population	Intervention, *n *	Smoking measure	Effect on smoking cessation, or prevalence
Smoking cessation							

Borland et al., 2009 [70]	Australia, Canada, UK, and US (ITC)	Longitudinal	2002–2006	Adults, age 18+	*Australia * (B) 6 rotating, text labels, 25% of front, 33% of back; (I) 14 rotating, graphic labels, 30% of front, 90% of back*, 2305; *Canada * (B) 16 rotating, graphic labels, 50% of pack, 2214; *UK * (B) 6 rotating, text labels, 6% of front; (I-1) 16 rotating, text labels, 30% of front, 40% of back; (I-2) banned use of “light”, “mild”, 2401; *US * (B) 4 rotating, text labels on side, 2138	Made a quit attempt lasting more than 24 hours since previous survey, and among those who did, whether quit attempt lasted at least 1 month	*Australia * F1 quit rate: 14.99% F2 quit rate: 22.93% F3 quit rate: 25.15% F4 quit rate: 25.90% *Canada * F1 quit rate: 19.84% F2 quit rate: 23.96% F3 quit rate: 22.81% F4 quit rate: 21.34% *United Kingdom * F1 quit rate: 16.83% F2 quit rate: 22.68% F3 quit rate: 28.93% F4 quit rate: 23.94% *United States * F1 quit rate: 14.42% F2 quit rate: 19.23% F3 quit rate: 20.31% F4 quit rate: 20.36%

Borland, 1997 [69]	Australia (original data collection)	Longitudinal	1994-1995	Adults, age 16+; 51% male	(B) 4 rotating, text-only labels covering 15% of front and back of package, 510; (I) 6 rotating, text-only labels covering 25% of front and 33% of back of package, 243	Quit smoking at followup for at least 1 week	Quit rate: 11%

Smoking prevalence							

Siahpush et al., 2009 [30]	Australia (Roy Morgan Single Source)	Before/after w/o comparison	1991–2006	Adults, age 18+; ages 18–29: 21%; ages 30–49: 41%; ages 50+: 38%; 48% male	(I) 6 rotating, text-only labels covering 25% of front and 33% of back of package, 515,866	Do you now smoke factory-made cigarettes? In the last month, have you smoked any roll-your-own cigarettes?	aRR = 1.00; *P* = 0.96

Gospodinov and Irvine, 2004 [71]	Canada (CTUMS)	Before/after w/o comparison	2000-2001	Adults, age 15+; 46% male	(B) text only, 9729; (I) 16 rotating, graphic labels, 50% of pack, 10447	Current cigarette smoking	Smoking prevalence: (B) 25.0% (I) 23.4% Marginal effect prevalence rate ratio: −0.0034 (95% CI: −0.029, 0.021; se = 0.01)

*Health warning label also included the quitline phone number.

aRR: adjusted rate ratio; B: baseline; CI: confidence interval; CTUMS: Canadian Tobacco Use Monitoring Surveys; F: followup period; I: intervention; ITC: International Tobacco Control Policy Research Survey; se: standard error; UK: United Kingdom; US: United States.

**Table 7:** Effects of antitobacco mass media campaigns on smoking initiation, cessation, and prevalence.

Author, year	Country (Data source)	Study design	Dates of data collection	Population	Intervention, *n *	Smoking measure	Effect on smoking initiation, cessation or prevalence
Smoking initiation							

Bauman et al., 1991 [145]	US (original data)	Cluster RCT	1985–1987	Youths, ages 12–14	(I) 8 30-second radio messages focused on 7 expected consequences of smoking broadcasted over 3 1-month periods; (C) no mass media campaign*, 951 total nonsmokers at baseline	Ever puffed a cigarette	Among nonsmokers at baseline, differences relative to comparison group at 11–17 months after broadcasts ended (i) Smoking experimentation: 1% (*P *= NS) (ii) Regular smoking: 2% (*P *= NS) (iii) Recent smoking: 1% (*P* = NS)

Farrelly et al., 2009 [75]	US (NLSY97)	Longitudinal study	1997–2004	Youths, ages 12–17	(I) TV campaign with cumulative exposure between 2000–2004 of 3096–32137 GRPs across 210 media markets, 8904	Ever smoked a cigarette	HR = 0.8 (95% CI: 0.71–0.91; *P* = 0.001) (per 10,000 GRP cumulative exposure)

Linkenbach and Perkins, 2003 [72]	US (original data)	Longitudinal study	2000–2001	Youths, junior and senior high school students; mean age = 14.6; 50% male	(I) 1500 GRPs (broadcast TV); 78,000 print and promotional items distributed in schools; 4 theater slides were run over 1 month at 2 movie theaters; 1 billboard design appeared in 4 locations for 1 month, 299; (C) control, 314	Having tried cigarette smoking	12-month follow-up smoking prevalence: (I) 10% (C) 17% Relative measure: 41% lower rate of initiation in intervention group (*P* < 0.05)

Flynn et al., 1997 [73]	US (original data)	Longitudinal study	1985–1991	Youths, grades 4–6	(I) 540 TV and 350 radio broadcasts per year for 4 years plus school intervention; (C) school intervention	Smoked >0 cigarettes in past week	4-year follow-up smoking prevalence: (I) 7.5% (C) 13.0% 6-year followup smoking prevalence: (I) 15.9% (C) 20.2% OR = 0.73

Hafstad et al., 1997 [74]	Norway (original data)	Longitudinal study	1992–1995	Youths, ages 14-15	(I) 3 annual campaigns of 1 TV and cinema ad 167 times, 3 full-page ads in 5 newspapers, 1 poster in each location run for 3 weeks; (C) control county	Weekly smoking	*Males * 1-year initiation rate (I) 10.2% (C) 14.5% OR = 0.67 (95% CI: 0.53; 0.85) *Females * 1-year initiation rate (I) 14.6% (C) 25.6% OR = 0.77 (95% CI: 0.63; 0.95)

Smoking cessation							

Solomon et al., 2009 [80]	US (original data)	Cluster RCT	2001–2004	Youths, grades 7–10; 45% male	(I) radio and TV campaign with 380 GRPs/week over 9 months each year for 3 years, 531; (C) no intervention, 601	Not smoking one cigarette in past 30 days	12-month quit rate (I) 18.1% (C) 14.8% 24-month quit rate (I) 14.5% (C) 12.6% 36-month quit rate (I) 16.0% (C) 12.8% Relative measure: no significant time trend or interaction between condition and time

Terry-McElrath et al., 2011 [81]	US (MTF)	Longitudinal	2001–2008	Adults, age 20–30	24-month sum of antitobacco TV advertising measured in GRPs, 7997	Smoked 0 cigarettes/day in past 30 days	*<52 GRPS* (ref) *52*–*103 GRPs * aOR: 1.15 (95% CI: 0.91; 1.45) *104*–*155 GRPs * aOR: 1.40 (95% CI: 1.07; 1.83) *156*–*207 GRPs * aOR: 1.21 (95% CI: 0.90; 1.63) *208*+ *GRPs * aOR: 1.22 (95% CI: 0.90; 1.66)

Burns and Levinson, 2010 [82]	US (original data collection)	Longitudinal	2007	Adults, age 18+ 41–50% male	(I) Spanish-language TV campaign with 1387.4 GRPs for 1 month, radio ads, and 1900 30-second spots on movie screens, 117 (C) non-Spanish speaking population, 193	6-month abstinence	Quit rate prior to campaign (I) 9.6% (C) 16.5% Quit rate post campaign (I) 18.8%; *P* < 0.05 (C) 8.8%; *P* = 0.01

Durkin et al., 2009 [146]	US (UMass Tobacco Study)	Longitudinal	2001–2004	Adults mean age = 41 45% male	24-month GRPs	1-month abstinence	Quit rate, 16%

Hyland et al., 2006 [147]	US (COMMIT)	Longitudinal study	1988–2001	Adults, ages 24–64	(I) TV campaign above 1218 GRPs in 1999-2000 (C) TV campaign below 1218 GRPs in 1999-2000	NR	24-month quit rate (I) 12.9% (C) 11.0% RR = 1.1 (95% CI: 0.98–1.24) (per increase in 5000 GRPs of exposure)

Ronda et al., 2004 [76]	Netherlands (original data)	Longitudinal study	1998–2001	Adults, ages 18+ 39–47% male; Mean age: 46–50 years	(I) Billboard, print, radio, TV, posters and postcards in waiting rooms and public buildings; 4 months spread over 2 years^†^	Not having smoking any tobacco in last 7 days	24-month quit rate (I) 12.3% (C) 14.3% 36-month quit rate (I) 18.7% (C) 18.6% relative measure: no association between intervention and smoking outcome in regression models (not reported)

McVey and Stapleton, 2000 [148]	England (original data)	Longitudinal study	1992–1994	Adults, ages 16+ 41-42% male; Mean age: 46 years	(I) 18-month TV campaign, 1744; (C) no intervention, 719	No smoking at all nowadays	18-month quit rate (I) 9.7% (C) 8.7% OR = 1.27 (95% CI: 0.77–2.08; *P* = 0.35)

Hafstad et al., 1997 [74]	Norway (original data)	Longitudinal study	1992–1995	Youths, ages 14-15	(I) 3 annual campaigns of 1 TV and cinema ad 167 times, 3 full-page ads in 5 newspapers, 1 poster in each location run for 3 weeks, 1061; (C) control county, 1288	Weekly smoking	*Males * 1-year quit rate (I) 12.7% (C) 19.1% OR = 0.63 *Females * 1-year quit rate (I) 25.6% (C) 17.6% OR = 1.9

Smoking prevalence							

Flynn et al., 2010 [83]	US (original data collection)	Cluster RCT	2001–2005	Youths, grades 7–12, 46% male	(I) 380 GRPs from TV ads per week, 215 GRPs from radio ads, 10,412; (C) no intervention, 9544	Smoking in past 30 days	Baseline smoking prevalence (I) 18.9% (C) 17.8% Smoking intervention at 4 year followup (I) 16.9% (C) 15.5%; *P* = 0.95

Wakefield et al., 2008 [27]	Australia (Roy Morgan Single Source)	Time series	1995–2006	Adults, age 18+	138-month TV campaign, 288.5 mean monthly GRPs, 343,835	Smoke factory-made cigarettes	Prevalence percentage point change two months later (i.e., 2 month lag) per 1 GRP per month increase:−0.00077 (95% CI: −0.00144, −0.0001; *P* = 0.025)

Hafstad et al., 1997 [74]	Norway (original data)	Longitudinal study	1992–1995	Youths, ages 14-15	(I) 3 annual campaigns of 1 TV and cinema ad 167 times, 3 full-page ads in 5 newspapers, 1 poster in each location run for 3 weeks, 2742; (C) control county, 3438	Weekly smoking	OR = 0.74 (95% CI: 0.64; 0.86) *Males * Baseline prevalence (I) 6.9% (C) 9.9% 1-year prevalence (I) 13.7% (C) 20.4% *Females * Baseline prevalence (I) 10.1% (C) 11.4% 1-year prevalence (I) 18.7% (C) 23.8%

Flynn et al., 1995 [149] Worden et al., 1996 [150] Flynn et al., 1992 [151] Flynn et al., 1994 [152] Worden and Flynn, 2002 [153] Flynn et al., 1997 [73]	US (original data)	Longitudinal study	1985–1991	Youths, grades 4–6; mean age: 10.6 years, 48–54% male	(I) 540 TV and 350 radio broadcasts per year for 4 years plus school intervention; (C) school intervention	Smoked >0 cigarettes in past week	Baseline prevalence (I) 1% (C) 1.6% 6-year prevalence (I) 16.5% (C) 24% OR = 0.62 (95% CI: 0.49; 0.78) *Females * 4-year prevalence (I) 12.7%; *P* < 0.01 (C) 21.1% 6-year prevalence (I) 16.5% (C) 29.4%; *P* < 0.01 *Males * 4-year prevalence (I) 9.8%;, *P* = 0.16 (C) 14.4% 6-year prevalence (I) 13.0% (C) 17.1%; *P* = 0.23

Steenkamp et al., 1991 [77]	South Africa (original data)	Longitudinal study	1979–1983	Adults, ages 15–64 46% male	(I) 48-month billboard, print, poster, and mailing campaign^‡^, 1531; (C) control, 1308	Smoking an average of at least 1 cigarette or 1 gram of tobacco per day	Baseline prevalence (I) 28.1% (C) 29.5% 4-year prevalence (I) 18.8% (C) 19.9% percentage Reduction (I) −32.6% (C) −33.3% Net percentage change in smoking prevalence relative to control Males: 2.0% Females: −19.2%

Meshack et al., 2004 [154]	US (original data)	Before/after with comparison	Spring 2000–December 2000	Youths, grade 6 52% male	(I) 3 × 3 media and community program; media programs involved TV, radio, billboard, and print; $0.50 per capita in low-intensity group; $1.00 per capita in high-intensity group, 3618	Tobacco use in past 30 days	Percent change in prevalence at 8.5 months (among groups with no community program): High intensity: −20.8% Low intensity: −45.3% Comparison: −28.3%

Sly et al., 2001 [79]	US (original data)	Before/after with comparison	1998-1999	Youths, ages 12–17	(I) 12-month campaign with TV, radio, billboard, display ads, promotional items (stickers, lanyards, hats, t-shirts, etc.), 1600 GRPs per quarter, 1800; (C) control, 1000	At least a puff or two in the past 30 days	Baseline prevalence (I) 13.8% (C) 12.6% 12-month prevalence (I) 12.6% (C) 14.1% Percentage change (I) −8.9% (C) 11.9% *P* < 0.05%

Farrelly et al., 2005 [78]	US (MTF)	Before/after w/o comparison	1997–2002	Youths, grades 8, 10, and 12	(I) 24-month TV campaign with 3867–20367 GRPs (cumulative exposure over 2-year period for the lowest and highest quintiles of exposure)	Any smoking in past 30 days	Percentage annual change in prevalence at 0–2 years *prior* to intervention: Total: −3.2% (−3.8, −2.6) 8th: −3.4% (−4.6, −2.1) 10th: −4.6% (−5.6, −3.6) 12th: −1.8% (−2.7, −1.0) Percentage annual change in prevalence at 0–2 years *after* intervention: Total: −6.8% (−7.5, −6.1) 8th: −9.0% (−10.4, −7.6) 10th: −8.7% (−9.8, −7.5) 12th: −5.1% (−6.1, −3.9)

*Additionally, there were 2 other intervention groups that included sweepstakes. Since sweepstakes are not a focus of this paper, they are not included here.

^†^This was part of a cardiovascular disease prevention campaign.

^‡^This was part of a coronary risk factor campaign.

C: control group; CI: confidence interval; COMMIT: Community Intervention Trial for Smoking Cessation; GRPs: gross rating points; HR: hazard ratio; I: intervention group; MTF: Monitoring the Future: a Continuing Study of American Youth; NLSY97: National Longitudinal Survey of Youth 1997; NR: not reported; NS: not significant; OR: odds ratio; RCT: randomized controlled trial; RR: relative risk; TV: television; US: United States.

## Appendix

### PubMed Search Strategies

The following Search Strings were used.

Search Number 1 ((“Smoking/epidemiology”[mh] OR “Smoking/prevention and control”[mh] OR “Smoking/economics”[mh] OR smoking[tiab] OR smoker*[tiab] OR smoked[tiab] OR cigarette*[tiab] OR tobacco[tiab] OR cigar[tiab] OR bidi*[tiab] OR hooka*[tiab] OR waterpipe*[tiab] OR kretek*[tiab] OR shisha*[tiab]) AND (price[tiab] OR prices[tiab] OR tax[tiab] OR taxes[tiab] OR taxation[tiab])) NOT (animals[mh] NOT humans[mh]).

Search Number 2 ((“Smoking/epidemiology”[mh] OR “Smoking/prevention and control”[mh] OR “Smoking/psychology”[mh] OR “Smoking/legislation and jurisprudence”[mh] OR smoking[tiab] OR smoker*[tiab] OR smoked[tiab] OR cigarette*[tiab] OR tobacco[tiab] OR cigar*[tiab] OR bidi*[tiab] OR hooka*[tiab] OR waterpipe*[tiab] OR kretek*[tiab] OR shisha*[tiab]) AND (((bars[tiab] OR pubs[tiab] OR (employee*[tiab] AND (polic*[tiab] OR program*[tiab])) OR indoor*[tiab] OR restaurant*[tiab] OR workplace*[tiab] OR work-place*[tiab] OR office*[tiab] OR hospital*[tiab]) AND (smoke-free[tiab] OR smokefree[tiab] OR “smoke free”[tiab] OR anti-smoking[tiab] OR antismoking[tiab] OR no-smoking[tiab] OR “no smoking”[tiab] OR non-smoking[tiab] OR nonsmoking[tiab] OR (smoking[tiab] AND employee*[tiab]) OR ban[tiab] OR bans[tiab] OR banning[tiab] OR law[tiab] OR legislation[tiab] OR prohibiti*[tiab] OR “smoking restriction”[tiab] OR “smoking restrictions”[tiab] OR “tobacco restriction”[tiab] OR ordinance*[tiab])) OR ((smoke-free[tiab] OR smokefree[tiab] OR “smoke free”[tiab] OR anti-smoking[tiab] OR antismoking[tiab] OR no-smoking[tiab] OR “no smoking”[tiab] OR non-smoking[tiab] OR nonsmoking[tiab] OR “smoking ban”[tiab] OR “smoking bans”[tiab]) AND (ban[tiab] OR bans[tiab] OR banning[tiab] OR law[tiab] OR legislation[tiab] OR prohibiti*[tiab] OR “smoking restriction”[tiab] OR “smoking restrictions”[tiab] OR ordinance*[tiab])))) NOT (animals[mh] NOT humans[mh]).

Search Number 3 ((“Smoking/epidemiology”[mh] OR “Smoking/prevention and control”[mh] OR “Smoking/psychology”[mh] OR “Smoking/legislation and jurisprudence”[mh] OR smoking[tiab] OR smoker*[tiab] OR smoked[tiab] OR cigarette*[tiab] OR tobacco[tiab] OR cigar*[tiab] OR bidi*[tiab] OR hooka*[tiab] OR waterpipe*[tiab] OR kretek*[tiab] OR shisha*[tiab]) AND ((advertis*[tiab] OR brand*[tiab] OR marketing[tiab] OR ordinance*[tiab] OR message*[tiab] OR television[tiab] OR tv[tiab] OR televised[tiab] OR “motion pictures”[tiab] OR radio[tiab] OR newspaper*[tiab] OR movie*[tiab] OR “in-store”[tiab] OR “in store”[tiab] OR magazine*[tiab] OR email[tiab] OR “e-mail”[tiab] OR internet[tiab] OR web[tiab] OR print[tiab] OR campaign*[tiab] OR commercial[tiab] OR commercials*[tiab] OR ((display[tiab] OR displays[tiab]) AND (retail[tiab] OR store[tiab] OR “point of purchase”[tiab] OR “point-of-purchase”[tiab OR “point of sale”[tiab] OR “point-of-sale”[tiab] OR “self-service”[tiab] OR “self service”[tiab] OR “self-serve”[tiab] OR “self serve”[tiab])) OR sponsor*[tiab]) AND ((adolescent*[tiab] OR children[tiab] OR minor*[tiab] OR teenager*[tiab] OR teens[tiab] OR “under-age”[tiab] OR young[tiab] OR youth*[tiab] OR kids[tiab]) OR (ban[tiab] OR bans[tiab] OR banning[tiab] OR law[tiab] OR laws[tiab] OR legislation*[tiab] OR sale[tiab] OR sales[tiab] OR purchas*[tiab] OR initiat*[tiab] OR behav*[tiab] OR restrict*[tiab] OR forbid*[tiab] OR prohibit*[tiab])))) NOT (animals[mh] NOT humans[mh]).

Search Number 4 ((“Smoking/epidemiology”[mh] OR “Smoking/prevention and control”[mh] OR “Smoking/psychology”[mh] OR “Smoking/legislation and jurisprudence”[mh] OR smoking[tiab] OR smoker*[tiab] OR smoked[tiab] OR cigarette*[tiab] OR tobacco[tiab] OR cigar*[tiab] OR bidi*[tiab] OR beedi*[tiab] OR hooka*[tiab] OR waterpipe*[tiab] OR kretek*[tiab] OR shisha*[tiab] OR chutta*[tiab] OR dhumti*[tiab] OR hookli*[tiab] OR chillum*[tiab]) AND ((health[tiab] AND (warning*[tiab] OR label*[tiab])) OR (warning*[tiab] AND label*[tiab]) OR ((mild[tiab] OR light[tiab] OR “low tar”[tiab]) AND (packs[tiab] OR packet*[tiab] OR package*[tiab] OR label*[tiab])) OR ((“mass media”[tiab] OR television[tiab] OR tv[tiab] OR televised[tiab] OR “motion pictures”[tiab] OR radio[tiab] OR newspaper*[tiab] OR movie*[tiab] OR “in-store”[tiab] OR “in store”[tiab] OR magazine*[tiab] OR email[tiab] OR “e-mail”[tiab] OR internet[tiab] OR web[tiab] OR print[tiab] OR advertis*[tiab] OR campaign*[tiab] OR promotion*[tiab] OR marketing[tiab] OR commercial*[tiab] OR packs[tiab] OR package*[tiab] OR packet*[tiab]) AND (initiat*[tiab] OR cessation[tiab] OR quit[tiab] OR quitting[tiab] OR stop[tiab] OR stopping[tiab] OR antismoking[tiab] OR “anti-smoking”[tiab] OR antitobacco[tiab] OR antitobacco[tiab])))) NOT (animals[mh] NOT humans[mh]).

Search Number 5 Number 1 OR Number 2 OR Number 3 OR Number 4.

## References

[1] Mathers CD, Loncar D (2006). Projections of global mortality and burden of disease from 2002 to 2030. *PLoS Medicine*.

[2] Jha P, Ranson MK, Nguyen SN, Yach D (2002). Estimates of global and regional smoking prevalence in 1995, by age and sex. *American Journal of Public Health*.

[3] Lopez AD, Collishaw NE, Piha T (1994). A descriptive model of the cigarette epidemic in developed countries. *Tobacco Control*.

[4] Health Organization World WHO Framework Convention on Tobacco Control. http://www.who.int/fctc/about/en/index.html.

[5] Health Organization World (2008). *WHO Report on the Global Tobacco Epidemic, 2008: The MPOWER Package*.

[6] for Healthcare Research and Quality Agency Methods Guide for Effectiveness and Comparative Effectiveness Reviews. http://www.effectivehealthcare.ahrq.gov/methodsguide.cfm.

[7] Nonnemaker JM, Farrelly MC (2011). Smoking initiation among youth: the role of cigarette excise taxes and prices by race/ethnicity and gender. *Journal of Health Economics*.

[8] Sen A, Wirjanto T (2010). Estimating the impacts of cigarette taxes on youth smoking participation, initiation, and persistence: empirical evidence from Canada.. *Health Economics*.

[9] Cawley J, Markowitz S, Tauras J (2004). Lighting up and slimming down: the effects of body weight and cigarette prices on adolescent smoking initiation. *Journal of Health Economics*.

[10] Tauras JA (2005). Can public policy deter smoking escalation among young adults?. *Journal of Policy Analysis and Management*.

[11] DeCicca P, Kenkel D, Mathios A (2002). Putting out the fires: will higher taxes reduce the onset of youth smoking?. *Journal of Political Economy*.

[12] Tauras JA, Chaloupka FJ Determinants of smoking cessation: an analysis of young adult men and women.

[13] Grossman M (2005). Individual behaviours and substance use: the role of price.. *Advances in Health Economics and Health Services Research*.

[14] Tauras JA, Chaloupka FJ Price, clean indoor air, cigarette smoking: evidence from longitudinal data for young adults.

[15] Kostova D, Ross H, Blecher E, Markowitz S (2011). Is youth smoking responsive to cigarette prices? Evidence from low- and middle-income countries. *Tobacco Control*.

[16] White VM, Warne CD, Spittal MJ, Durkin S, Purcell K, Wakefield MA (2011). What impact have tobacco control policies, cigarette price and tobacco control programme funding had on Australian adolescents’ smoking? Findings over a 15-year period. *Addiction*.

[17] Carpenter C, Cook PJ (2008). Cigarette taxes and youth smoking: new evidence from national, state, and local Youth Risk Behavior Surveys. *Journal of Health Economics*.

[18] Ding A (2003). Youth are more sensitive to price changes in cigarettes than adults. *Yale Journal of Biology and Medicine*.

[19] Waller BJ, Cohen JE, Ferrence R, Bull S, Adlaf EM (2003). The early 1990s cigarette price decrease and trends in youth smoking in Ontario. *Canadian Journal of Public Health*.

[20] Gruber J Youth smoking in the US: prices and policies.

[21] Chaloupka FJ, Pacula RL Limiting youth access to tobacco: the early impact of the synar amendment on youth smoking.

[22] Ross H, Blecher E, Yan L, Hyland A (2011). Do cigarette prices motivate smokers to quit? New evidence from the ITC survey. *Addiction*.

[23] Saenz-de-Miera B, Thrasher JF, Chaloupka FJ, Waters HR, Hernandez-Avila M, Fong GT (2010). Self-reported price of cigarettes, consumption and compensatory behaviours in a cohort of Mexican smokers before and after a cigarette tax increase. *Tobacco control*.

[24] Hanewinkel R, Isensee B (2007). Five in a row - Reactions of smokers to tobacco tax increases: population-based cross-sectional studies in Germany 2001–2006. *Tobacco Control*.

[25] Franz GA (2008). Price effects on the smoking behaviour of adult age groups. *Public Health*.

[26] Reed MB, Anderson CM, Vaughn JW, Burns DM (2008). The effect of cigarette price increases on smoking cessation in California. *Prevention Science*.

[27] Wakefield MA, Durkin S, Spittal MJ (2008). Impact of tobacco control policies and mass media campaigns on monthly adult smoking prevalence. *American Journal of Public Health*.

[28] Azagba S, Sharaf M (2011). Cigarette taxes and smoking participation: evidence from recent tax increases in Canada. *International Journal of Environmental Research and Public Health*.

[29] Lance PM, Akin JS, Dow WH, Loh CP (2004). Is cigarette smoking in poorer nations highly sensitive to price? Evidence from Russia and China. *Journal of Health Economics*.

[30] Siahpush M, Wakefield MA, Spittal MJ, Durkin SJ, Scollo MM (2009). Taxation reduces social disparities in adult smoking prevalence. *American Journal of Preventive Medicine*.

[31] DeCicca P, McLeod L (2008). Cigarette taxes and older adult smoking: evidence from recent large tax increases. *Journal of Health Economics*.

[32] Franks P, Jerant AF, Leigh JP (2007). Cigarette prices, smoking, and the poor: implications of recent trends. *American Journal of Public Health*.

[33] Sloan FA, Trogdon JG (2004). The impact of the master settlement agreement on cigarette consumption. *Journal of Policy Analysis and Management*.

[34] Gallus S, Fernandez E, Townsend J, Schiaffino A, La Vecchia C (2003). Price and consumption of tobacco in Italy over the last three decades. *European Journal of Cancer Prevention*.

[35] Scollo M, Younie S, Wakefield M, Freeman J, Icasiano F (2003). Impact of tobacco tax reforms on tobacco prices and tobacco use in Australia. *Tobacco Control*.

[36] van Walbeek C (2002). Recent trends in smoking prevalence in South Africa—some evidence from AMPS data. *South African Medical Journal*.

[37] Farrelly MC, Bray JW, Pechacek T, Woollery T (2001). Response by adults to increases in cigarette prices by sociodemographic characteristics. *Southern Economic Journal*.

[38] Bogdanovica I, Murray R, McNeill A, Britton J (2012). Cigarette price, affordability and smoking prevalence in the European Union. *Addiction*.

[39] Jimenez-Ruiz JA, Saenz de Miera B, Reynales-Shigematsu LM, Waters HR, Hernandez-Avila M (2008). The impact of taxation on tobacco consumption in Mexico. *Tobacco Control*.

[40] Hu TW, Mao Z, Shi J, Chen W (2010). The role of taxation in tobacco control and its potential economic impact in China. *Tobacco Control*.

[41] Hawkins SS, Cole TJ, Law C (2011). Examining smoking behaviours among parents from the UK millennium cohort study after the smoke-free legislation in Scotland. *Tobacco Control*.

[42] Klein EG (2008). The unintended consequences of clean indoor air policies in Minnesota. *Dissertation Abstracts International B*.

[43] Biener L, Hamilton WL, Siegel M, Sullivan EM (2010). Individual, Social-normative, and policy predictors of smoking cessation: a multilevel longitudinal analysis. *American Journal of Public Health*.

[44] Hyland A, Hassan LM, Higbee C (2009). The impact of smokefree legislation in Scotland: results from the Scottish ITC Scotland/UK longitudinal surveys. *European Journal of Public Health*.

[45] Bauza-Amengual MdeL, Blasco-Gonzalez M, Sanchez-Vazquez E, Pereiro-Berenguer I, Ruiz-Varea N, Pericas-Beltran J (2010). Impact of the Tobacco Law on the workplace: a follow up study of a cohort of workers in Spain 2005–2007. *Aten Primaria*.

[46] Murphy JM, De Moreno SL, Cummings KM, Hyland A, Mahoney MC (2010). Changes in cigarette smoking, purchase patterns, and cessation-related behaviors among low-income smokers in New York state from 2002 to 2005. *Journal of Public Health Management and Practice*.

[47] Orbell S, Lidierth P, Henderson CJ (2009). Social-cognitive beliefs, alcohol, and tobacco use: a prospective community study of change following a ban on smoking in public places. *Health Psychology*.

[48] Martinez-Sanchez JM, Fernandez E, Fu M (2009). Impact of the Spanish smoking law in smoker hospitality workers. *Nicotine & Tobacco Research*.

[49] Fowkes FJI, Stewart MCW, Fowkes FGR, Amos A, Price JF (2008). Scottish smoke-free legislation and trends in smoking cessation. *Addiction*.

[50] De Chaisemartin C, Geoffard PY, Le Faou AL (2011). Workplace smoking ban effects on unhappy smokers. *Health Economics*.

[51] Hahn EJ, Rayens MK, Ridner SL, Butler KM, Zhang M, Staten RR (2010). Smoke-free laws and smoking and drinking among college students. *Journal of Community Health*.

[52] Hahn EJ, Rayens MK, Butler KM, Zhang M, Durbin E, Steinke D (2008). Smoke-free laws and adult smoking prevalence. *Preventive Medicine*.

[53] Lemstra M, Neudorf C, Opondo J (2008). Implications of a public smoking ban. *Canadian Journal of Public Health*.

[54] Klein EG, Forster JL, Erickson DJ, Lytle LA, Schillo B (2009). The relationship between local clean indoor air policies and smoking behaviours in minnesota youth. *Tobacco Control*.

[55] Gallus S, Zuccaro P, Colombo P (2006). Effects of new smoking regulations in Italy. *Annals of Oncology*.

[56] Guerrero F, Santonja FJ, Villanueva RJ (2011). Analysing the Spanish smoke-free legislation of 2006: a new method to quantify its impact using a dynamic model. *International Journal of Drug Policy*.

[57] Mackay DF, Haw S, Pell JP (2011). Impact of Scottish smoke-free legislation on smoking quit attempts and prevalence. *PLoS One*.

[58] Mullally BJ, Greiner BA, Allwright S, Paul G, Perry IJ (2009). The effect of the Irish smoke-free workplace legislation on smoking among bar workers. *European Journal of Public Health*.

[59] Galan I, Mata N, Estrada C (2007). Impact of the “Tobacco control law” on exposure to environmental tobacco smoke in Spain. *BMC Public Health*.

[60] Haw SJ, Gruer L (2007). Changes in exposure of adult non-smokers to secondhand smoke after implementation of smoke-free legislation in Scotland: National Cross sectional survey. *British Medical Journal*.

[61] Lee JT, Glantz SA, Millett C (2011). Effect of smoke-free legislation on adult smoking behaviour in England in the 18 months following implementation. *PLoS One*.

[62] Elton PJ, Campbell P (2008). Smoking prevalence in a north-west town following the introduction of Smoke-free England. *Journal of Public Health*.

[63] Anger S, Kvasnicka M, Siedler T (2011). One last puff? Public smoking bans and smoking behavior. *Journal of Health Economics*.

[64] Verdonk-Kleinjan WM, Candel MJ, Knibbe RA, Willemsen MC, de Vries H (2011). Effects of a workplace-smoking ban in combination with tax increases on smoking in the Dutch population. *Nicotine & Tobacco Research*.

[65] Bitler MP, Carpenter C, Zavodny M (2011). Smoking restrictions in bars and bartender smoking in the US, 1992–2007. *Tobacco Control*.

[66] Bitler MP, Carpenter CS, Zavodny M (2010). Effects of venue-specific state clean indoor air laws on smoking-related outcomes. *Health Economics*.

[67] Galduróz JCF, Fonseca AM, Noto AR, Carlini EA (2007). Decrease in tobacco use among Brazilian students: a possible consequence of the ban on cigarette advertising?. *Addictive Behaviors*.

[68] Fielding R, Chee YY, Choi KM (2004). Declines in tobacco brand recognition and ever-smoking rates among young children following restrictions on tobacco advertisements in Hong Kong. *Journal of Public Health*.

[69] Borland R (1997). Tobacco health warnings and smoking-related cognitions and behaviours. *Addiction*.

[70] Borland R, Yong HH, Wilson N (2009). How reactions to cigarette packet health warnings influence quitting: findings from the ITC Four-Country survey. *Addiction*.

[71] Gospodinov N, Irvine IJ (2004). Global health warnings on tobacco packaging: evidence from the Canadian experiment. *Topics in Economic Analysis and Policy*.

[72] Linkenbach JW, Perkins HW, Perkins HW (2003). MOST of us are tobacco free: an eight-month social norms campaign reducing youth initiation of smoking in Montana. *The Social Norms Approach to Preventing School and College Age Substance Abuse: A Handbook for Educators, Counselors, and Clinicians*.

[73] Flynn BS, Worden JK, Secker-Walker RH, Pirie PL, Badger GJ, Carpenter JH (1997). Long-term responses of higher and lower risk youths to smoking prevention interventions. *Preventive Medicine*.

[74] Hafstad A, Aarø LE, Engeland A, Andersen A, Langmark F, Stray-Pedersen B (1997). Provocative appeals in anti-smoking mass media campaigns targeting adolescents—the accumulated effect of multiple exposures. *Health Education Research*.

[75] Farrelly MC, Nonnemaker J, Davis KC, Hussin A (2009). The influence of the National Truth campaign on smoking initiation. *American Journal of Preventive Medicine*.

[76] Ronda G, Van Assema P, Candel M (2004). The Dutch Heart Health Community Intervention “Hartslag Limburg”: effects on smoking behaviour. *European Journal of Public Health*.

[77] Steenkamp HJ, Jooste PL, Jordaan PCJ, Swanepoel ASP, Rossouw JE (1991). Changes in smoking during a community-based cardiovascular disease intervention programme. The Coronary Risk Factor Study. *South African Medical Journal*.

[78] Farrelly MC, Davis KC, Haviland ML, Messeri P, Healton CG (2005). Evidence of a dose-response relationship between “truth” antismoking ads and youth smoking prevalence. *American Journal of Public Health*.

[79] Sly DF, Heald GR, Ray S (2001). The Florida “truth” anti-tobacco media evaluation: design, first year results, and implications for planning future state media evaluations. *Tobacco Control*.

[80] Solomon LJ, Bunn JY, Flynn BS, Pirie PL, Worden JK, Ashikaga T (2009). Mass media for smoking cessation in adolescents. *Health Education and Behavior*.

[81] Terry-McElrath YM, Emery S, Wakefield MA, O’Malley PM, Szczypka G, Johnston LD Effects of tobacco-related media campaigns on smoking among 20–30-year-old adults: longitudinal data from the USA.

[82] Burns EK, Levinson AH (2010). Reaching spanish-speaking smokers: state-level evidence of untapped potential for quitLine utilization. *American Journal of Public Health*.

[83] Flynn BS, Worden JK, Bunn JY (2010). Mass media interventions to reduce youth smoking prevalence. *American Journal of Preventive Medicine*.

[84] Arunatilake N (2002). An economic analysis of tobacco demand in Sri Lanka. *Sri Lanka Economic Journal*.

[85] Chaloupka FJ, Hu T, Warner KE, Jacobs R, Yurekli A, Jha P, Chaloupka F (2000). The taxation of tobacco products. *Tobacco Control in Developing Countries*.

[86] (2009). *IARC Handbooks of Cancer Prevention, Tobacco Control, Vol. 13: Evaluating the effectiveness of smoke-free policies*.

[87] of Medicine Institute (2009). *Secondhand Smoke Exposure and Cardiovascular Effects: Making Sense of the Evidence*.

[88] Warner K (2006). *Tobacco Control Policy*.

[89] Department of Health and Human Services, Public Health Service Centers for Disease Control and Prevention US (1994). *Preventing tobacco use among young people: a report of the Surgeon General*.

[90] Cancer Institute National (Bethesda, Md, USA). *Smoking and Tobacco Control Monograph 14*.

[91] DiFranza JR, Wellman RJ, Sargent JD, Weitzman M, Hipple BJ, Winickoff JP (2006). Tobacco promotion and the initiation of tobacco use: assessing the evidence for causality. *Pediatrics*.

[92] Brown A, Moodie C (2009). The influence of tobacco marketing on adolescent smoking intentions via normative beliefs. *Health Education Research*.

[93] Wellman RJ, Sugarman DB, DiFranza JR, Winickoff JP (2006). The extent to which tobacco marketing and tobacco use in films contribute to children’s use of tobacco: a meta-analysis. *Archives of Pediatrics and Adolescent Medicine*.

[94] Saffer H, Chaloupka F (2000). The effect of tobacco advertising bans on tobacco consumption. *Journal of Health Economics*.

[95] Hammond D, Fong GT, McNeill A, Borland R, Cummings KM (2006). Effectiveness of cigarette warning labels in informing smokers about the risks of smoking: Findings from the International Tobacco Control (ITC) Four Country Survey. *Tobacco Control*.

[96] Hammond D, Fong GT, Borland R, Cummings KM, McNeill A, Driezen P (2007). Text and graphic warnings on cigarette packages: findings from the international tobacco control four country study. *American Journal of Preventive Medicine*.

[97] of Medicine Institute (2007). *Ending the Tobacco Problem: A Blueprint for the Nation*.

[98] Thrasher JF, Hammond D, Fong GT, Arillo-Santillán E (2007). Smokers’ reactions to cigarette package warnings with graphic imagery and with only text: a comparison between Mexico and Canada. *Salud Publica de Mexico*.

[99] Hammond D, McDonald PW, Fong GT, Brown KS, Cameron R (2004). The impact of cigarette warning labels and smoke-free bylaws on smoking cessation: evidence from former smokers. *Canadian Journal of Public Health*.

[100] White V, Webster B, Wakefield M (2008). Do graphic health warning labels have an impact on adolescents’ smoking-related beliefs and behaviours?. *Addiction*.

[101] Hammond D, Fong GT, McDonald PW, Cameron R, Brown KS (2003). Impact of the graphic Canadian warning labels on adult smoking behaviour. *Tobacco Control*.

[102] Fathelrahman AI, Omar M, Awang R (2009). Smokers’ responses toward cigarette pack warning labels in predicting quit intention, stage of change, and self-efficacy. *Nicotine and Tobacco Research*.

[103] Cancer Institute National (2008). *The Role of the Media in Promoting and Reducing Tobacco Use*.

[104] Wakefield M, Flay B, Nichter M, Giovino G (2003). Role of the media in influencing trajectories of youth smoking. *Addiction*.

[105] Farrelly MC, Niederdeppe J, Yarsevich J (2003). Youth tobacco prevention mass media campaigns: past, present, and future directions. *Tobacco Control*.

[106] Farrelly MC, Healton CG, Davis KC, Messeri P, Hersey JC, Haviland ML (2002). Getting to the truth: evaluating national tobacco countermarketing campaigns. *American Journal of Public Health*.

[107] Hopkins DP, Briss PA, Ricard CJ (2001). Reviews of evidence regarding interventions to reduce tobacco use and exposure to environmental tobacco smoke (Structured abstract). *American Journal of Preventive Medicine*.

[108] Bala M, Strzeszynski L, Cahill K (2008). Mass media interventions for smoking cessation in adults.. *Cochrane Database of Systematic Reviews*.

[109] Cancer Institute National (2006). *Evaluating ASSIST: A Blueprint for Understanding State-level Tobacco Control*.

[110] Frieden TR, Mostashari F, Kerker BD, Miller N, Hajat A, Frankel M (2005). Adult tobacco use levels after intensive tobacco control measures: New York City, 2002-2003. *American Journal of Public Health*.

[111] Department of Public Health and California Tobacco Control Program California California Tobacco Control Update 2009: 20 Years of Tobacco Control in California.

[112] Levy DT, Mumford EA, Gerlowski DA (2007). Examining trends in quantity smoked. *Nicotine and Tobacco Research*.

[113] Sung HY, Hu TW, Ong M, Keeler TE, Sheu ML (2005). A major state tobacco tax increase, the master settlement agreement, and cigarette consumption: the California experience. *American Journal of Public Health*.

[114] Stehr M (2005). Cigarette tax avoidance and evasion. *Journal of Health Economics*.

[115] (1998). Response to increases in cigarette prices by race/ethnicity, income, and age groups—United States, 1976–1993. *Morbidity and Mortality Weekly Report*.

[116] Meier KJ, Licari MJ (1997). The effect of cigarette taxes on cigarette consumption, 1955 through 1994. *American Journal of Public Health*.

[117] Hu TW, Sung HY, Keeler TE (1995). Reducing cigarette consumption in California: tobacco taxes vs an anti-smoking media campaign. *American Journal of Public Health*.

[118] Keeler TE, Hu TW, Barnett PG, Manning WG (1993). Taxation, regulation, and addiction: a demand function for cigarettes based on time-series evidence. *Journal of Health Economics*.

[119] Flewelling RL, Kenney E, Elder JP, Pierce J, Johnson M, Bal DG (1992). First-year impact of the 1989 California cigarette tax increase on cigarette consumption. *American Journal of Public Health*.

[120] Peterson DE, Zeger SL, Remington PL, Anderson HA (1992). The effect of state cigarette tax increases on cigarette sales, 1955 to 1988. *American Journal of Public Health*.

[121] Hu TW, Sung HY, Keeler TE (1995). The state antismoking campaign and the industry response: the effects of advertising on cigarette consumption in California. *The American economic review*.

[122] Sung H-Y, Hu T-W, Keeler TE (1994). Cigarette taxation and demand: an empirical model. *Contemporary Economic Policy*.

[123] Keeler TE, Hu TW, Manning WG, Sung HY (2001). State tobacco taxation, education and smoking: controlling for the effects of omitted variables. *National Tax Journal*.

[124] Keeler TE, Hu TW, Ong M, Sung HY (2004). The US national Tobacco Settlement: the effects of advertising and price changes on cigarette consumption. *Applied Economics*.

[125] Baltagi BH, Levin D (1992). Cigarette taxation: raising revenues and reducing consumption. *Structural Change and Economic Dynamics*.

[126] Blecher E (2008). The impact of tobacco advertising bans on consumption in developing countries. *Journal of Health Economics*.

[127] Stewart MJ (1993). The effect on tobacco consumption of advertising bans in OECD countries. *International Journal of Advertising*.

[128] Guindon E, Perucic AM, Boisclair D Higher tobacco prices and taxes in South-east Asia: an effective tool to reduce tobacco use, save lives and generate revenue.

[129] Farrelly MC, Nimsch CT, Hyland A, Cummings M (2004). The effects of higher cigarette prices on tar and nicotine consumption in a cohort of adult smokers. *Health Economics*.

[130] Gruber J, Sen A, Stabile M (2003). Estimating price elasticities when there is smuggling: the sensitivity of smoking to price in Canada. *Journal of Health Economics*.

[131] Galbraith JW, Kaiserman M (1997). Taxation, smuggling and demand for cigarettes in Canada: evidence from time-series data. *Journal of Health Economics*.

[132] Mummery WK, Hagen LC (1996). Tobacco pricing, taxation, consumption and revenue: Alberta 1985–1995. *Canadian Journal of Public Health*.

[133] Reinhardt FS, Giles DEA (2001). Are cigarette bans really good economic policy?. *Applied Economics*.

[134] Bardsley P, Olekalns N (1999). Cigarette and tobacco consumption: have anti-smoking policies made a difference?. *Economic Record*.

[135] Szilágyi T (2007). Higher cigarette Taxes—healthier people, wealthier state: the Hungarian experience. *Central European Journal of Public Health*.

[136] Hanewinkel R, Radden C, Rosenkranz T (2008). Price increase causes fewer sales of factory-made cigarettes and higher sales of cheaper loose tobacco in Germany. *Health Economics*.

[137] Lee JM, Liao DS, Ye CY, Liao WZ (2005). Effect of cigarette tax increase on cigarette consumption in Taiwan. *Tobacco Control*.

[138] Fernandez E, Gallus S, Schiaffino A (2004). Price and consumption of tobacco in Spain over the period 1965–2000. *European Journal of Cancer Prevention*.

[139] Borren P, Sutton M (1992). Are increases in cigarette taxation regressive?. *Health economics*.

[140] Chapman S, Richardson J (1990). Tobacco excise and declining tobacco consumption: the case of Papua New Guinea. *American Journal of Public Health*.

[141] Mao ZZ, Xiang JL, Kon ZP (1997). Demand for cigarette and pricing policy. *Chinese Health Economics*.

[142] Djutaharta T, Viriya-Surya H, Haidy N, Pasay A, Moertiningsih-Adioetomo H, Moertiningsih-Adioetomo S Aggregate analysis of the impact of cigarette tax rate increases on tobacco consumption and governement revenue. The case of Indonesia.

[143] Michie S, van Stralen MM, West R (2011). The behaviour change wheel: a new method for characterising and designing behaviour change interventions. *Implementation Science*.

[144] Gospodinov N, Irvine I (2009). Tobacco taxes and regressivity. *Journal of Health Economics*.

[145] Bauman KE, LaPrelle J, Brown JD, Koch GG, Padgett CA (1991). The influence of three mass media campaigns on variables related to adolescent cigarette smoking: results of a field experiment. *American Journal of Public Health*.

[146] Durkin SJ, Biener L, Wakefield MA (2009). Effects of different types of antismoking ads on reducing disparities in smoking cessation among socioeconomic subgroups. *American Journal of Public Health*.

[147] Hyland A, Wakefield M, Higbee C, Szczypka G, Cummings KM (2006). Anti-tobacco television advertising and indicators of smoking cessation in adults: a cohort study. *Health Education Research*.

[148] McVey D, Stapleton J (2000). Can anti-smoking television advertising affect smoking behaviour? Controlled trial of the health education authority for England’s anti-smoking TV campaign. *Tobacco Control*.

[149] Flynn BS, Worden JK, Secker-Walker RH, Badger GJ, Geller BM (1995). Cigarette smoking prevention effects of mass media and school interventions targeted to gender and age groups. *Journal of Health Education*.

[150] Worden JK, Flynn BS, Solomon LJ, Secker-Walker RH, Badger GJ, Carpenter JH (1996). Using mass media to prevent cigarette smoking among adolescent girls. *Health Education and Behavior*.

[151] Flynn BS, Worden JK, Secker-Walker RH, Badger GJ, Geller BM, Costanza MC (1992). Prevention of cigarette smoking through mass media intervention and school programs. *American Journal of Public Health*.

[152] Flynn BS, Worden JK, Secker-Walker RH (1994). Mass media and school interventions for cigarette smoking prevention: effects 2 years after completion. *American Journal of Public Health*.

[153] Worden JK, Flynn BS, Hornik RC (2002). Using mass media to prevent cigarette smoking. *Public Health Communication: Evidence for Behavior Change*.

[154] Meshack AF, Hu S, Pallonen UE, McAlister AL, Gottlieb N, Huang P (2004). Texas tobacco prevention pilot initiative: processes and effects. *Health Education Research*.

